# The Role of Inter- and Intraspecific Variations in Grassland Plant Functional Traits along an Elevational Gradient in a Mediterranean Mountain Area

**DOI:** 10.3390/plants10020359

**Published:** 2021-02-13

**Authors:** Letizia Di Biase, Simone Fattorini, Maurizio Cutini, Alessandro Bricca

**Affiliations:** 1 Department of Life, Health and Environmental Sciences, University of L’Aquila, Via Vetoio, 67100 L’Aquila, Italy; letizia.dibiase@graduate.univaq.it; 2 Department of Science, University of Roma Tre, Viale G. Marconi 446, 00146 Roma, Italy; 3 School of Biosciences and Veterinary Medicine, University of Camerino, via Pontoni 5, 62032 Camerino, Italy; alessandro.bricca@unicam.it

**Keywords:** community nonweighted mean, community-weighted mean, functional traits, plant height, seed mass, specific leaf area, stress gradient

## Abstract

Elevational gradients offer special opportunities to investigate the relative role of intraspecific and interspecific trait variations in relation to stress gradients. We used an altitudinal gradient in the Mediterranean (Mt Velino, Central Italy) to study (1) how community-weighted means (CWM) and nonweighted means (CM) vary with elevation for plant height, specific leaf area, and seed mass; and (2) how variation patterns differ for inter- and intraspecific functional variability. We tested (1) if elevation influences community functional composition on the basis of the adaptive value of plant traits and (2) if the latter shows intraspecific variations according to the species’ ability to cope with local conditions. We found that different traits showed different patterns, which can be linked to the function they express. Differences between communities were influenced more by differences between their traits (CM) than by the relative species coverage (CWM). Both highest and lowest elevations were the most selective due to their particularly severe climatic conditions. Intermediate elevations were the most favorable thanks to less constraining climatic conditions. Interspecific trait variability was the most relevant component, indicating a low plant ability to cope with environmental variations through phenotypic plasticity.

## 1. Introduction

There is an increasing interest in the use of plant functional traits to understand how vegetation responds to environmental factors [1,2,3]. Plant traits are quantitative descriptors of species ecological niche, being directly linked to species’ fitness and providing information on resource uses and habitat requirements [4]. One of the most widely used metrics to investigate how environmental conditions affect plant communities is the community-weighted mean (CWM), which is calculated as the sum of trait values of the species in an assemblage, weighted by their relative abundance [5]. This metric is indicative of the selective effects of the environment on the community [5,6,7], as the traits of the dominant species are those that play the major role in determining the response of the plant community to the environment [8].

In addition to CWM, another frequently used metric to express mean trait values at community level is the so-called community nonweighted mean (CM), which corresponds to the mean of the trait values for the species not weighted for their relative abundance [9]. As CM is not determined by the species abundances, but exclusively by the presence or absence of the species and the values of their traits, comparing CM and CWM can be useful to highlight the potential role of the subdominant species in the studied community [9,10,11].

Most functional investigations have considered the trait values as fixed for each species, assuming that trait differences are much larger between species than within species [12], i.e., that intraspecific variation in trait measures is absent or negligeable [12]. Although many traits are very conservative and show low or no plasticity (for example, the metabolic pathways for carbon and nitrogen fixation), most quantitative traits exhibit significant intraspecific variations [4,13,14,15].

Recent studies have demonstrated that the intraspecific trait variability in response to environmental factors is much greater than expected [14,16,17,18] and might be important in determining community composition and ecosystem functioning [19,20,21]. Thus, at the community level, three sources of variation can be recognized [22]: (i) species turnover, i.e., species composition (and possibly species relative cover) may change but trait values are constant within each species; (ii) intraspecific trait variability, i.e., trait values change, even if species cover or species identity remain the same; and (iii) covariation between turnover and intraspecific variability. Assessing the relative contribution of intra- and interspecific variation in the traits considered, and the direction of the shifts in their values (covariation), may prove particularly useful in modelling community responses to changes in environmental parameters [11,16,23,24]. Both CWM and CM can be used to investigate these three sources of variability (interspecific, intraspecific and covariation), but while CWM expresses variations in both species identity and species cover, CM considers only the species identity.

In this context, elevational gradients offer a special opportunity to investigate how communities and their sources of variation may respond to environmental parameters, because in mountain areas environmental characteristics (especially the climatic ones) may change even within a small spatial scale [25,26], markedly affecting plant traits [24,27]. In the Mediterranean mountains, plants are subject to stressful conditions not only at higher elevations because of lower temperatures [28], but also at the lowest elevations due to the scarcity of precipitation, especially in the summer period [29], leading to the so-called “double stress” gradient, with the predominance of winter frost stress at higher elevation and summer drought stress condition at lower elevation [30]. Thus, elevational gradients are particularly suitable for studying how changes in stressing conditions drive plant morphological variability.

Since functional traits reflect the way in which plants vary their ecological strategies of growth, the use of resources and their response to the environment [31], it is expected that plant communities living along an elevational gradient should show a strong variation in functional trait dominance in response to stress gradients. From this perspective, investigating the contribution of intraspecific and interspecific trait variability to the total community functional composition may be important because it helps disentangle the influence of factors operating at larger scales (which are more important in determining interspecific trait variation through the selective effects of the stressors on species adaptations) from that of factors which operate on a local scale (which are expected to more profoundly influence the intraspecific variation) [12].

Variations in the intraspecific component of traits can occur in the same direction of the interspecific component (positive covariation) or in the opposite direction (negative covariation) [11,22]. Using the mean trait values calculated along the entire environmental gradient, the CWM can show a certain relationship of interspecific variation with the given environmental parameter. The intraspecific responses in plants, though, could reveal an opposite trend (negative covariation). In this case, if the two sources of variation have a similar extent, the total community variation may not show patterns at all. If the intra- and interspecific variations have the same direction (case of a positive covariation), then the total community variation will be very pronounced [22].

Finally, it has been noted that the relative contribution of inter- vs. intraspecific trait variation to shifts in community-average trait values following environmental changes reflects the resistance of plant community structure to these changes, with higher relative interspecific variation representing lower resistance [12,32]. A higher relative importance of intraspecific trait variability in relation to environmental variation in CM reflects greater resistance of plant community composition to environmental changes [33]. In contrast, a higher relative contribution of intraspecific trait variation in CWM indicates greater resistance of the plant community structure, that is, the composition and relative abundance of the dominant species [16].

In this paper, we used the vegetation present along an elevational gradient in the Mediterranean area to study (1) how the weighted (CWM) and nonweighted (CM) functional composition of plant communities vary in response to elevation for different functional traits and (2) how the variation patterns differ for inter- and intraspecific variability. Our final aim was to test if climatic stressors associated with elevation (namely high aridity at lower elevations and low temperature and frost at higher elevations) not only influence which species compose each community on the basis of the adaptive values of their traits, but also if the latter show more or less pronounced intraspecific variations in different environmental contexts according to the ability of the species to adapt to local conditions.

To this end, we considered three quantitative traits to illustrate plant species functions in the communities: vegetative maximum height (H), specific leaf area (SLA) and seed mass (SM). These traits are referred to the leaf–height–seed scheme [34], which has proved to be a useful integrated framework to capture key plant ecological strategies [35]. H is related to competitive ability in light-limited environments and aboveground resource acquisition [36], but can be constrained by water availability, with shorter plants growing in colder conditions [37]. SLA is related to resource exploitation underlying a trade-off between acquisitive and conservative strategies, with lower values indicating slow-growing species and higher values indicating fast-growing species [38,39]. SLA is known to decrease with decreasing temperatures [40]. SM variation expresses a species’ chance of successfully dispersing a seed and a seedling’s ability to survive various hazards [40]. It seems there is no clear, ubiquitous pattern of variation in seed mass along elevational gradients [38]. On one hand, smaller seeds might be selected at high altitudes due to the shortness of the growing season; on the other hand, larger seeds might be selected as they might be more resistant in habitats characterized by frost and water stress.

## 2. Results

### 2.1. Maximum Height (H)

**Figure 1:**
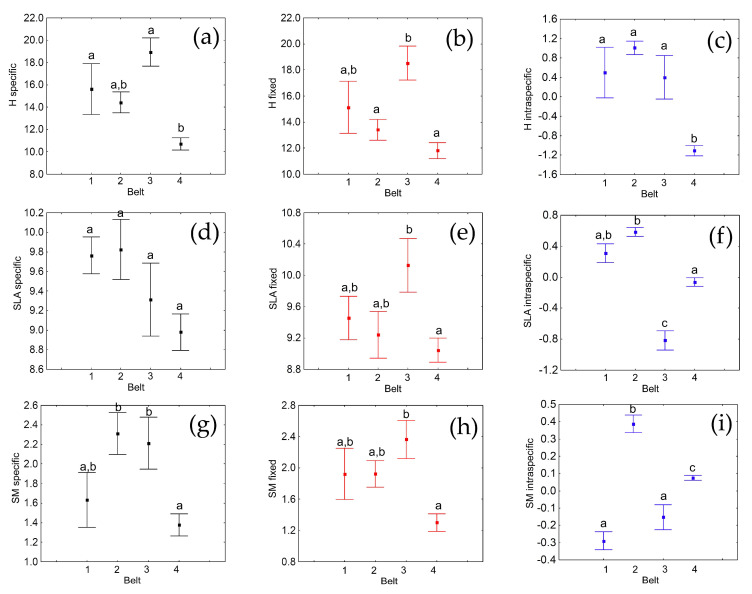
Averages and standard errors of community-weighted mean (CWM) values in the four elevational belts (elevation: 1, 2, 3, 4) calculated for plant height (H: **a**, **b**, **c**), specific leaf area (SLA: **d**, **e**, **f**) and seed mass (SM, **g**, **h**, **i**), along an elevational gradient in Central Italy. CWMs were calculated for the specific variability (**a**, **d**, **g**), the fixed component (**b**, **e**, **h**) and the intraspecific variability (**c**, **f**, **i**). Letters indicate Tukey honest significant difference (HSD) post-hoc tests at the *p* < 0.05 level, following ANOVA.

Both fixed and specific CWM averages varied significantly between belts (Figure 1a,b, Table 1A). Post hoc tests for specific values demonstrated significant differences between the first and the fourth belts and between the third and the fourth belts (Table 1A). In particular, the specific CWM values were, on average, significantly lower in the fourth belt than in the first and third belts (Figure 1a). Fixed CWM values (which express compositional turnover) varied significantly between the second and the fourth belts and between the third and the fourth belts; the difference between the second and third belts was marginally not significant (*p* = 0.07) (Table 1A). The fourth belt had the lowest mean value for the fixed CWM (Figure 1b). Variation in intraspecific CWM values between belts was also globally significant, with post hoc tests showing significant differences between the fourth belt (which showed the lowest value) and all the others (Figure 1c, Table 1A,). Hence, the fourth belt was the belt that differed more from the others, but such differences arose from different components. While the differences with the first and the third belts were due to both fixed and intraspecific variability of H, the difference with the second belt was limited to intraspecific variability. Furthermore, the difference between the second and the third belts was essentially due to species turnover, because if the intraspecific variability is considered, the difference becomes marginally nonsignificant. Decomposition of total variability showed that variability among belts caused by turnover was almost twelve times higher than that caused by intraspecific variation (Table 1B). Furthermore, these results showed how altitude explained only a small fraction of both the intraspecific variability (about 58% remained unexplained) and the compositional (fixed) variability (67% not explained) (Table 1C). The variability in specific CWM values was further increased by a positive covariation between turnover and intraspecific variability effects (Table 1C).

**Figure 2:**
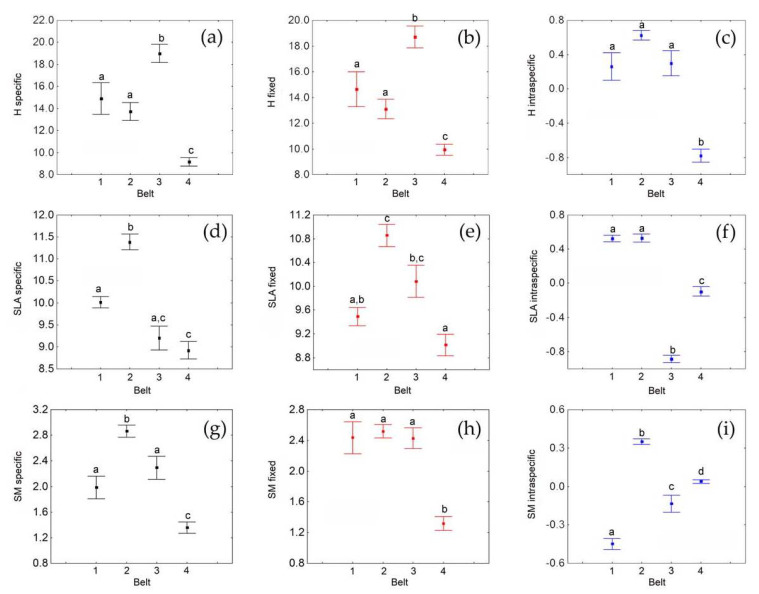
Averages and standard errors of community nonweighted mean (CM) values in the four elevational belts (elevation: 1, 2, 3, 4) calculated for plant height (H: **a**, **b**, **c**), specific leaf area (SLA: **d**, **e**, **f**) and seed mass (SM, **g**, **h**, **i**), along an elevational gradient in Central Italy. CMs were calculated for the specific variability (**a**, **d**, **g**), the fixed component (**b**, **e**, **h**) and the intraspecific variability (**c**, **f**, **i**). Letters indicate Tukey HSD post-hoc tests at the *p* < 0.05 level, following ANOVA.

Using the CM, both fixed and specific CM average values varied significantly between belts (Figure 2a,b, Table 2A). Post hoc tests for specific values demonstrated significant differences in all pairwise comparisons, except between the first and the second belts (Table 2A). In particular, both the specific CM and the fixed values were, on average, highest in the third belt and lowest in the fourth belt (Figure 2a). Intraspecific variation between belts was also globally significant, with post hoc tests showing significant differences between the fourth belt (which showed the lowest value) and all the others (Figure 2c, Table 2A). Hence, the fourth belt was the belt that differed more from the others for all components (fixed, specific and intraspecific), whereas the third belt had higher values than the others for the fixed and specific components, but not for the intraspecific one. Decomposition of total variability showed that variability among belts caused by turnover was more than 26 times higher than that caused by intraspecific variation (Table 2B). Furthermore, these results showed how altitude explained only a small fraction of the intraspecific variability (about 53% remained unexplained) but a larger fraction of the compositional variability (38% not explained) (Table 2C). The variability in specific averages was further increased by a positive covariation between turnover and intraspecific variability effects (Table 2C).

### 2.2. Specific Leaf Area (SLA)

Fixed CWM average values varied significantly between belts, whereas variation in the specific values was not significant (*p* = 0.09), although a certain decrease with increasing elevation was apparent (Figure 1d,e, Table 3A). Post hoc tests for the fixed values demonstrated that the fourth belt had a significantly lower average than the third belt (Table 3A). Intraspecific variations between belts were globally significant, with post hoc tests being significant for all pairwise comparisons, except for the comparison involving the first and the second belts (Table 3A). The fourth and the third belts had significantly lower CWM values than the first and second belts, and the average CWM value of the third belt was significantly lower than that of the fourth belt (Figure 1f, Table 3A). This indicates that belts differed mostly in SLA intraspecific variability, whereas the fixed component was important only for the difference between the third and the fourth belts. Decomposition of total variability showed that variability among belts caused by turnover was almost three times higher than that caused by intraspecific variation (Table 3B). Furthermore, these results show how altitude explained a relatively large fraction of the intraspecific variability (about 24% remained unexplained), but the compositional (fixed) variability was poorly explained (80% not explained) (Table 3C). We found a negative covariation between turnover and intraspecific variability effects (Table 3C), as CWM was higher in the third than in the fourth belt for the fixed component but was higher in the fourth than in the third belt for the intraspecific component, which can explain the lack of a significant result for the ANOVA conducted on specific values.

Using CM, both fixed and specific CM average values varied significantly between belts (Figure 2d,e, Table 4A). Post hoc tests for specific values demonstrated significant differences in all pairwise comparisons, except between the first and the third belts, which had a marginally nonsignificant difference (*p* = 0.07), and between the third and the fourth belts (Table 4A). Post hoc tests for fixed values demonstrated significant differences in all pairwise comparisons, except between the first and the third belts, between the first and the fourth belts and between the second and the third belts, which had a marginally nonsignificant difference (*p* = 0.07) (Table 4A). Intraspecific variation between belts was also globally significant, with post hoc tests showing significant differences in all pairwise comparisons, except between the first and the second belts (Figure 2f, Table 4A). The third and the fourth belts had lower average values in comparison with the first and the second, this latter showing the highest average value (Figure 2d). Fixed values were higher in the second and third belts (Figure 2c). The third belt showed the lowest average for the intraspecific variation (Figure 2f). In this belt, turnover and intraspecific variability showed opposite effects, which can explain why the fourth belt did not differ for the specific component. Decomposition of total variability showed that variability among belts caused by turnover was about three times higher than that caused by intraspecific variation (Table 4B). Furthermore, altitude explained a large fraction of the intraspecific variability (only about 7% remained unexplained), whereas the compositional variability was poorly explained (47% not explained) (Table 4C). The variability in specific averages was slightly increased by a positive covariation between turnover and intraspecific variability effects (Table 4C).

### 2.3. Seed Mass (SM)

Both fixed and specific CWM average values varied significantly between belts (Figure 1g,h, Table 5A). Post hoc tests for specific values demonstrated significant differences between the second and the fourth belts and between the third and the fourth belts (Table 5A). In particular, the specific CWM values were, on average, significantly lower in the fourth belt than in the second and third belts, but similar to those of the first belt (Figure 1g). Fixed CWM values varied significantly between the third and the fourth belts, which showed the lowest average (Table 5B, Figure 1h). Intraspecific variation between belts was also globally significant, with post hoc tests showing significant differences in all pairwise comparisons, except between the first and the third belts (Figure 1i, Table 5A). The second belt had the highest average, followed by the fourth belt; the first and third belts had the lowest values. This indicates that belts differed mostly in SM intraspecific variability, whereas the fixed component was important only for the difference between the third and the fourth belts. Thus, the fourth belt was that mostly differing from the others, but the different components acted in a contrasting way. The fourth belt showed a higher average of the intraspecific variability (Figure 1i) but lower averages for the specific (Figure 1g) and fixed (Figure 1h) effects. Decomposition of total variability showed that variability among belts caused by turnover was more than seven times higher than that caused by intraspecific variation (Table 5B), which explains why the fourth belt had a significantly lower CWM for the specific effect despite the highest intraspecific effect. Altitude explained a relatively large fraction of the intraspecific variability (about 29% remained unexplained), but the compositional variability was poorly explained (about 79% not explained) (Table 5C). We found a negative covariation between turnover and intraspecific variability effects (Table 5C), which can explain the contrasting results for the fourth belt.

Using CM, both fixed and specific CM average values varied significantly between belts (Figure 2g,h, Table 6A). Post hoc tests for specific values demonstrated significant differences in all cases except between the first and the third belts (Table 6A). Fixed CM values varied significantly between the fourth belt and all the others (Table 6A). Intraspecific variation between belts was also globally significant, with post hoc tests showing significant differences in all pairwise comparisons (Figure 2i, Table 6A). The fourth belt had the lowest averages for both the specific (Figure 2g) and the fixed (Figure 2h) effects, whereas the fourth belt had a higher average than the first and the third belts for the intraspecific component (Figure 2i). This indicates that belts differed mostly in SM intraspecific variability, whereas the fixed component was important only for the difference between the third and the fourth belts. Thus, the fourth belt was that mostly differing from the others, but the different components acted in a contrasting way. The fourth belt showed a higher average for the intraspecific variability (Figure 2i), but lower averages for the specific (Figure 2g) and fixed (Figure 2h) effects. Decomposition of total variability showed that the variability among belts caused by turnover was more than five times higher than that caused by intraspecific variation (Table 6B), which explains why the fourth belt had a significantly lower CM average for the specific effect despite the highest intraspecific effect. Altitude explained a very large fraction of the intraspecific variability (less than 3% remained unexplained), but the compositional variability was poorly explained (about 57% not explained) (Table 6C). We found a negative covariation between turnover and intraspecific variability effects (Table 6C), which can explain the contrasting results for the fourth belt.

## 3. Discussion

### 3.1. Variations in CWM and CM Values

Variations in vegetation characteristics along elevational gradients are mainly determined by climatic factors, in particular by decreasing temperatures [25,26,28]. However, other factors, such as rainfall patterns, topography, nutrient availability and anthropogenic impacts, can contribute to shaping vegetation at high altitudes [28,41]. The study of how the functional structure of plant communities varies along elevational gradients has attracted much attention [23,24,29,42,43,44,45,46,47,48,49], yet most of the available studies are limited to the analysis of single traits [50]. Kichenin et al. [23], for example, showed that the leaf area tends to decrease with increasing altitude in most species, which is reflected in decreasing CWM values. However, variation in the functional composition of plant communities can be adequately understood only if several traits are examined simultaneously [51,52]. Furthermore, almost nothing is known for the elevational gradients in the Mediterranean basin [3,53].

Most of the studies that used CWM and CM have focused on interspecific variability, thus overlooking the potential impact of intraspecific variability [16,54]. However, intraspecific variability can contribute substantially to the overall response of functional traits to environmental gradients [16,55,56]. In our study, the use of CWM highlighted that interspecific variation was the predominant source of variability in all cases (albeit with a nonsignificant effect for SLA), a pattern in agreement with previous findings [23]. This means that the stressful factors that vary with altitude exert their influence essentially in terms of species composition and relative abundances, thus operating on a coarse scale. After all, the main factors affecting plant species along the elevational gradients are the climatic ones (in particular, variations in temperatures and precipitation), which are expected to overwhelm those operating at the local level (e.g., possible local differences between plots of the same belt).

In all cases, the lowest CWM specific and fixed values were found in the fourth belt, which therefore appears to be that with the most stressing conditions (especially because of frost periods). The species occurring in the fourth belt showed adaptations to cope with low temperatures and frost stress, such as shorter size (a lower height allows species to benefit from soil heat) [25,42,57], leaves with smaller surface per unit of dry mass (a phenomenon observed at high altitudes as an adaptation to frost) [25,50] and smaller seeds (a common condition at high altitude due to the short growing season) [57].

Although less relevant than the interspecific component in terms of the amount of variability explained, the intraspecific variability exerted a significant role in all cases. For all studied traits (H, SLA and SM), highest intraspecific CWM averages were recorded in the second belt, which suggests that this is the most favorable altitude for plant life, as both drought stress and frost stress are not intense here [29]. Indeed, low CWM values observed in the first belt could be a consequence of plant adaptation to summer water stress [58]. In the second belt, drought stress might be mitigated by the lowering of temperatures due to the increase in altitude. At even higher elevations, in the third and fourth belts, environmental conditions become more hostile, especially because of the particularly low temperatures [29].

For H, the lowest value of intraspecific CWM and CM observed in the fourth belt suggests that the frost stress affected the plant community by selecting not only shorter species [25,27] but also shorter individuals (as a positive covariation between inter- and intraspecific variability pointed out). In fact, differences in terms of plant height between the first three belts were minimal, which suggests a strong local selection on the plant height only in conditions of intense frost/low-temperature stress. A reduction in plant height is typically observed in conditions of high stress due to low temperatures; as soil temperature is higher than that of the air, shorter plants benefit more from soil heat [25,57]. Furthermore, when the ground is frozen, lower plants tend to be more protected from drying out by the snow cover [39]. For SLA and SM, on the other hand, the third belt showed lower values than the fourth.

SLA is a trait representative of strategies of the acquisition, use and storage of resources [59] and has been observed to decrease with decreasing temperatures [40]. The size of the seed (SM), on the other hand, expresses the ability of a species to colonize and develop seedlings [40]. Thus, our results indicate that high-elevation environments favor plants with more efficient strategies to exploit the few available resources and thus to cope with stressful conditions. It is also interesting to note that for the total community variability (i.e., the specific variability), SLA showed a decreasing trend with altitude. Indeed, lower SLA values could be an advantage in the presence of lower temperatures, as lower SLA values are related to a higher content of proteins and secondary compounds (such as proline) per leaf unit, which increases the resistance of leaves to freezing [60]. With regard to SM, larger seed sizes have been interpreted as an adaptation to increase the likelihood of reproductive success in hostile environments [61]. In our case, if species characterized by small seeds tend to be selected at high altitudes (due to the shortness of the growing season), on the other hand, among these species with small seeds, individuals with larger seeds might be selected as they have a higher reproductive success (which explains the negative covariation).

Variation in CM due to turnover (in this case, exclusively linked to the species identity, since the relative abundances are not taken into account in CM calculation) was the predominant source of variability in all cases, mirroring the patterns found for CWMs. For H, the altitude showed a moderate effect on intraspecific variability, but was relatively important in interspecific variability (turnover), whereas in SLA and SM, altitude explained a higher fraction of intraspecific variability, but a small fraction of the variability associated with turnover. These differences are attributable to the fact that the average CM for the fixed effect is associated with a greater variability than that of the average CM calculated with the intraspecific variability. For all considered traits (H, SLA and SM), both the specific and the fixed CM values had the lowest values in the fourth belt, as also observed for CWM.

In all considered traits, CM patterns of variation in the intraspecific component and in total variability were similar to those discussed for the CWM. Therefore, it appears that when the effect of variability due to variation in relative coverage is excluded, results tend to remain very similar. This indicates that the differences in the functional structure are essentially affected by differences in the traits between the communities, not by differences in species relative coverage. This result, combined with the fact that intraspecific and fixed CWM values showed different patterns of variation, suggests that there is a combination of mechanisms which regulate local diversity [62,63,64] and drive CWM variation on a larger scale. One possible explanation is that species with trait values that diverge most from the CWM (resulting subdominant) may nevertheless persist locally if stressors act more strongly on the phenotype as a whole than on individual traits [63,65,66].

### 3.2. Community Resistance and Covariation

The contribution of interspecific variation to the total variability of a trait in a community reflects its resistance to environmental variation: when the interspecific variation exceeds the intraspecific variation, the community has low resistance [12,32]. More specifically, when all species are weighted equally (i.e., when using CM), a greater importance of intraspecific variability in relation to environmental variation reflects a greater resistance of the plant community composition to environmental changes [33]. Conversely, when weighted averages (CWM) are used, a greater relative contribution of intraspecific variation indicates greater resilience of the community structure, reflecting the influence of both the identity and the relative abundance of dominant species [16,67].

In our case, variation explained by the intraspecific component was generally much lower than that of the interspecific component for both the CWM and the CM, which means that the investigated communities showed a low resistance to abiotic stresses, in terms of both species composition (CM) and dominance (CWM). This can be interpreted as a consequence of the severity of the stressors forcing communities to respond to such changes by reshaping themselves in a profound way.

Comparing the covariation between inter- and intraspecific variability can provide important insights into the relationships between community structure and environmental changes. Parallel trends (i.e., a positive covariation) may have additional effects that accentuate community responses [22,68]. Conversely, opposite trends (i.e., a negative covariation) between inter- and intraspecific variability can lead to a reciprocal compensation and thus to the lack of responses in the specific mean values [18,22]. Although the reasons behind negative covariation remain elusive, investigating the causes of negative covariation is essential for understanding plant community structure [16].

Using the CWM, we found positive covariation in H and negative covariations in SLA and SM. Using the CM, covariation was positive in H and SLA but negative for SM, where, however, it had a very minor role. In general, a positive covariation is expected when environmental factors favoring plant species with certain values of a given trait also favor the same traits at the level of individuals, thus reinforcing the community response in the same functional direction [22,69,70]. For example, soil fertility favors both taller species and, within these species, taller individuals [22]. The positive covariation observed in CWM values for H suggests that local factors act in concert with those operating at a coarser scale by selecting small individuals within small species in the fourth belt. Conversely, negative covariations in SLA and SM indicate that these traits are under the effect of a double environmental action: after being selected following one direction at the species level, communities are influenced in the opposite way at the individual level. A negative covariation for SLA has already been observed by Kichenin et al. [23], and it explains the absence of an effect of elevation on the total community variation already observed in another study [29]. While changes in temperature, amount of light, concentration of nutrients and water availability may explain the interspecific decrease in SLA at higher altitudes, the concomitant increase in terms of the intraspecific component is less obvious and must be interpreted according to the specific context [23]. In our case, the third belt is that with the highest average for the fixed component (i.e., it is the belt where the plants with the largest specific leaf surface were selected), but it is also that with the lowest intraspecific average (i.e., individuals responded locally by reducing their leaf surface). This suggests that since the plants prevailing in the third belt are those with already medium–high SLA, they do not need to have local individuals with a higher SLA, which may explain the negative covariation for this trait. Finally, the positive covariations found with CM values indicate that in general, stressful factors acting at the individual level operate in concert with those acting at the species level in terms of trait selection, and that contrasting responses are due to differences in species coverage, as highlighted by the CWM.

## 4. Materials and Methods

### 4.1. Study Area and Data Collection

The study was conducted on Mount Velino (maximum elevation 2486 m), a limestone massif located in Central Italy. The Velino massif exhibits a sub-Mediterranean bioclimate, with a summer drought period more marked at lower elevations and winter frost stress more marked at higher elevation [29]. During the growing season (May–September), at 1200 m, the mean temperature is 17 °C and precipitation is around 350 mm; at 2250 m, mean temperature is 8 °C and precipitation is 470 mm.

Sampling was done using 45 plots (2 m × 2 m) distributed along an elevational gradient from 1325 to 2375 m, which was divided into four elevational belts of equal extent (250 m) on the basis of the topographical and climatic setting: (1) 1325–1575 m (9 plots), (2) 1575–1825 m (10 plots), (3) 1825–2075 m (11 plots) and (4) 2075–2375 m (15 plots) (Table S1). All plots were located on open calcareous grasslands not affected by domestic grazing and were similar in their aspect and slope. Herbaceous species composition and relative cover were recorded during the growing season (May to August) in 2016 [29]. As measuring traits for all species in each plot would have been prohibitively laborious, we used a parsimonious approach [22] that considered only the dominant species (i.e., those representing altogether at least the 80% of the total vegetation cover in each belt) [29] as representative of the functional composition of the local communities [71]. Individuals collected for trait measurement were sampled in the middle of each belt.

We measured 15 different individuals for each species in each belt for plant height (cm) and 10 leaves of different individuals per species in each belt for SLA (mm^2^/mg). Leaves were scanned with a CanonScan LiDE 210 (Canon Inc., Tokyo, Japan), and the leaf area was calculated using WinDias software (Delta-T Devices Ltd., Cambridge, UK). To measure SM (mg), we collected at least 2 seeds from at least 3 individuals per species per belt. Further details on the study area and sampling procedures can be found in Bricca et al. [29]. We collected trait data for 11 species in the first belt, 10 species in the second belt, 12 species in the third belt and 17 species in the fourth belt (Tables S1–S3). All traits were collected according to standardized protocols of trait measurements [72].

For each species, two types of means were calculated for each trait: the overall mean (i.e., the arithmetic mean with all belts pooled) and the belt means (i.e., the arithmetic mean specific of each belt). The first mean is fixed, i.e., it does not take into account intraspecific variation along the gradient, and it is therefore called the “fixed” trait mean. The second mean varies according to the belt, and hence expresses the intraspecific variation along the gradient. As this mean is specific to each belt, it is called the “specific” trait mean (note that “specific” refers to belt specificity, not to the species). For each trait, specific average values (i.e., the averages calculated for each belt separately) are shown in Table S2, while fixed averages (i.e., the averages calculated over the entire gradient) are given in Table S3.

### 4.2. Data Analysis

For analytical purposes, we considered each plot as a sampling unit describing the local community. We used both the weighted (CWM) and the nonweighted (CM) community averages, as they provide two complementary types of information. Because the averages weighted by species abundance are driven by the functional identity of the most abundant species [61], CWM reflects the role of dominant species in the community structure, thus assuming that ecosystem processes are strongly influenced by the functional traits of dominant species in a community. However, because CWM values are strongly influenced by trait values of the most dominant species [5,73], they are also linked to the sampling or selection effects associated with the greater chance of including highly productive species in more diverse communities [74,75]. By contrast, CM averages are driven by only species presence or absence at the community level and are therefore influenced by only species composition. Thus, comparing CWM and CM is particularly useful for understanding the effects of dominant (with CWM) and nondominant species (with CM) [9,10,11] in response to environmental changes.

CWM was calculated for each trait in each plot as:

(1) $$\mathrm{CWM}=\sum_{i=1}^{s}p_{i}x_{i},$$

where *S* is the total number of species, *p*_i_ is the relative cover (weight) of species *i* and *x*_i_ is the trait value of species *i*.

In CM, only species presences/absences were considered, which meant that all species had the same weight. CM was therefore calculated for each trait in each plot as:

(2) $$\mathrm{CM}\;=\;\frac{\sum_{i=1}^{S}x_{i}}{S}.$$

Most of the studies using CWM and CM considered one trait value for each species for all community samples where the species occurs, i.e., a single trait value (i.e., a fixed value) calculated as the mean for the whole studied gradient (which corresponds to our overall “fixed” mean calculated across the entire gradient, as mentioned above). To take into account intraspecific trait variability, it has been proposed to use trait values specific for different environmental conditions, i.e., calculated using measures taken under different environmental conditions [22], which in our case, corresponds to the mean specific to each belt.

To assess the relative contribution of intra- and interspecific trait variability effects on both CWM and CM along the elevational gradient, we followed the approach proposed by Lepš et al. [22]. This method is based on the calculation, for each plot, of the “fixed” and “specific” community trait means. Specific CWM (and CM) values were calculated using the species trait values recorded in each specific belt (i.e., for each belt, the arithmetic mean of the values measured from the individuals of that specific belt). Thus, each species received, for each trait, a measure which varied among belts. The resulting variations in CWM (or CM) values represented differences in the overall functional community composition due to the intraspecific trait variation, species turnover and covariation [22]. By contrast, fixed CWM (or CM) values were calculated using, for each trait, the mean value calculated pooling measured individuals across all belts. This way, a species received, for each trait, only a single value, and the resulting CWM (or CM) values contained only the contribute of species composition to the total community variation. For any given belt, the intraspecific trait variability could be therefore calculated as the difference between the specific and the fixed CWM (or CM) values.

To decompose species turnover, intraspecific trait variation and their covariation, we used the ANOVA procedure proposed by Lepš et al. [22], in which the total sum of squares (SSspecific) of the trait variance related to an environmental variable (here elevation) is partitioned into ‘fixed’ (SSfixed), ‘intraspecific’ (SSintraspecific) and ‘covariation’ (SScov) effects, so that SSspecific = SSfixed + SSintraspecific + SScov.

To compare the differences in CWM and CM values between the four elevations, ANOVAs were followed by Tukey’s honest significant difference (HSD) post hoc pairwise comparisons (*p* < 0.05) [37,76]. There is no agreement about the log-transformation of trait measures before analysis. Because high trait values would have a greater influence on the arithmetic mean, making them more prone to sampling error [77], some authors [23] apply a log-transformation to all functional traits before their use. However, the fact that a certain species has a particularly high value for a certain trait may be ecologically important, and downweighting it with a logarithmic transformation may obscure this importance. Other authors log-transformed only certain traits [40,78,79] or seem to have not considered this problem [11,41,76]. We performed all ANOVAs by using both untransformed and log_10_-transformed values for all traits.

All calculations were made in the R environment [80]. CWM and CM values were calculated using the package FD [81]. For decomposition of the total SS, we used the function flexanova proposed by Lepš et al. [22] and available in the package cati [82].

CWM and CM values, expressing the specific, fixed and intraspecific variability, calculated with original and log-transformed values, are reported for each trait (H, SLA and SM) in Tables S4–S7. In the Results section, we present the ANOVA results related to each trait for each of the three types of CWM and CM (fixed, specific and intraspecific) using untransformed data. For both CWM and CM, use of log_10_-transformed traits produced results very similar to those obtained with untransformed values (Tables S8–S13, Figures S1 and S2) and were therefore not discussed in detail.

## 5. Conclusions

Our research highlighted how plant communities along an altitudinal gradient in a Mediterranean mountain context show important variations in their functional structure depending on the altitude as an effect of stressful conditions (represented by thermal and water stress factors), which identified intermediate elevations (second belt: 1575–1825 m) as the most favorable and the highest elevations (fourth belt: 2075–2375 m) as the most selective. This can be attributed to the fact that high-elevation environments are the most stressful due to their particularly severe climate (low temperature and frost periods). Intermediate elevations are most favorable even compared to the lowest, since the latter are characterized by a greater stress represented by summer aridity. Different traits, however, show different patterns, which can be linked to their function. In general, comparisons between intra- and interspecific variability showed that the latter was the most important component. This demonstrates a low ability of plants of high altitudes to cope with environmental variations through phenotypic plasticity.

## Figures and Tables

**Table 1:** Results of ANOVAs for CWM of plant height (H). (**A**) ‘Fixed’ and ‘specific’ averages and intraspecific variability effect were analyzed separately (one-way ANOVA), followed by post hoc (HSD) tests for pairwise comparisons. Note that the sum of squares (SS) corresponds to the amount of variability. DF = degrees of freedom, MS = mean of squares, F = Fisher’s F, *p* = probability. (**B**) Variability of individual components of trait variation (turnover, intraspecific and their covariation) between belts. Note that turnover, intraspecific variability and total variation are identical to SS columns in the ANOVA for fixed traits, intraspecific variability and specific traits, respectively. Covariation was obtained by subtracting the first two columns from the last. (**C**) Proportions of variability of individual components and their parts explained by elevation (belt). Note that the matrix was obtained from matrix B by dividing all of its elements by total SS for total variation, i.e., for specific traits.

**(A)**															
	**Fixed CWM**					**Specific CWM**					**Intraspecific CWM**				
	**SS**	**DF**	**MS**	**F**	***p***	**SS**	**DF**	**MS**	**F**	***p***	**SS**	**DF**	**MS**	**F**	***p***
Belt	302.300	3	100.767	6.713	<0.001	443.900	3	147.967	8.831	<0.001	32.620	3	10.873	9.766	<0.0001
Residuals	615.400	41	15.010			687.000	41	16.756			45.650	41	1.113		
Post hoc (HSD) tests						Post hoc (HSD) tests					Post hoc (HSD) tests				
Belt pairwise comparisons	*p*					Belt pairwise comparisons	*p*				Belt pairwise comparisons	*p*			
1 vs. 2	0.767					1 vs. 2	0.920				1 vs. 2	0.693			
1 vs. 3	0.226					1 vs. 3	0.290				1 vs. 3	0.998			
1 vs. 4	0.190					1 vs. 4	0.033				1 vs. 4	0.005			
2 vs. 3	0.022					2 vs. 3	0.072				2 vs. 3	0.550			
2 vs. 4	0.741					2 vs. 4	0.131				2 vs. 4	<0.0001			
3 vs. 4	<0.001					3 vs. 4	<0.0001				3 vs. 4	0.004			
**(B)**															
	**Turnover**					**Intraspecific variability**					**Covariation**			**Total**	
Belt	302.300					32.620					108.980			443.900	
Residuals	615.400					45.650					25.950			687.000	
Total	917.700					78.270					134.930			1130.900	
**(C)**															
	**Turnover**					**Intraspecific variability**					**Covariation**			**Total**	
Belt	0.267					0.029					0.096			0.393	
Residuals	0.544					0.040					0.023			0.607	
Total	0.811					0.069					0.119			1.000	

**Table 2:** Results of ANOVAs for CM of plant height (H). (**A**) ‘Fixed’ and ‘specific’ averages and intraspecific variability effect were analyzed separately (one-way ANOVA), followed by post hoc (HSD) tests for pairwise comparisons. (**B**) Variability of individual components of trait variation (turnover, intraspecific and their covariation) between belts. (**C**) Proportions of variability of individual components and their parts explained by elevation (belt). For details, see Table 1.

**(A)**															
	**Fixed CM**					**Specific CM**					**Intraspecific CM**				
	**SS**	**DF**	**MS**	**F**	***p***	**SS**	**DF**	**MS**	**F**	***p***	**SS**	**DF**	**MS**	**F**	***p***
Belt	499.300	3	166.433	22.770	<0.0001	631.600	3	210.533	28.220	<0.0001	14.580	3	4.860	35.130	<0.0001
Residuals	299.700	41	7.310			305.900	41	7.461			15.670	41	0.382		
Post hoc (HSD) tests						Post hoc (HSD) tests					Post hoc (HSD) tests				
Belt pairwise comparisons	*p*					Belt pairwise comparisons	*p*				Belt pairwise comparisons	*p*			
1 vs. 2	0.601					1 vs 2	0.781				1 vs 2	0.163			
1 vs. 3	0.009					1 vs 3	0.010				1 vs 3	0.996			
1 vs. 4	<0.001					1 vs 4	<0.0001				1 vs 4	<0.0001			
2 vs. 3	<0.001					2 vs 3	<0.001				2 vs 3	0.204			
2 vs. 4	0.032					2 vs 4	0.001				2 vs 4	<0.0001			
3 vs. 4	<0.0001					3 vs 4	<0.0001				3 vs 4	<0.0001			
**(B)**															
	**Turnover**					**Intraspecific variability**					**Covariation**			**Total**	
Belt	499.300					14.580					117.720			631.600	
Residuals	299.700					15.670					−9.470			305.900	
Total	799.000					30.250					108.250			937.500	
**(C)**															
	**Turnover**					**Intraspecific variability**					**Covariation**			**Total**	
Belt	0.533					0.016					0.126			0.674	
Residuals	0.320					0.017					−0.010			0.326	
Total	0.852					0.032					0.115			1.000	

**Table 3:** Results of ANOVAs for CWM of specific leaf area (SLA). (**A**) ‘Fixed’ and ‘specific’ averages and intraspecific variability effect were analyzed separately (one-way ANOVA), followed by post hoc (HSD) tests for pairwise comparisons. (**B**) Variability of individual components of trait variation (turnover, intraspecific and their covariation) between belts. (**C**) Proportions of variability of individual components and their parts explained by elevation (belt). For details, see Table 1.

**(A)**															
	**Fixed CWM**					**Specific CWM**					**Intraspecific CWM**				
	**SS**	**DF**	**MS**	**F**	***p***	**SS**	**DF**	**MS**	**F**	***p***	**SS**	**DF**	**MS**	**F**	***p***
Belt	7.990	3	2.663	3.458	0.025	5.760	3	1.920	2.335	0.088	11.650	3	3.883	43.390	<0.0001
Residuals	31.580	41	0.770			33.730	41	0.823			3.670	41	0.090		
Post hoc (HSD) tests						Post hoc (HSD) tests					Post hoc (HSD) tests				
Belt pairwise comparisons	*p*					Belt pairwise comparisons	*p*				Belt pairwise comparisons	*p*			
1 vs. 2	0.95					1 vs. 2	0.999				1 vs. 2	0.204			
1 vs. 3	0.335					1 vs. 3	0.682				1 vs. 3	<0.0001			
1 vs. 4	0.68					1 vs. 4	0.184				1 vs. 4	0.252			
2 vs. 3	0.111					2 vs. 3	0.571				2 vs. 3	<0.0001			
2 vs. 4	0.945					2 vs. 4	0.118				2 vs. 4	<0.0001			
3 vs. 4	0.017					3 vs. 4	0.792				3 vs. 4	<0.0001			
**(B)**															
	**Turnover**					**Intraspecific variability**					**Covariation**			**Total**	
Belt	7.990					11.650					−13.880			5.760	
Residuals	31.580					3.670					−1.520			33.730	
Total	39.570					15.320					−15.400			39.490	
**(C)**															
	**Turnover**					**Intraspecific variability**					**Covariation**			**Total**	
Belt	0.202					0.295					−0.351			0.146	
Residuals	0.800					0.093					−0.038			0.854	
Total	1.000					0.388					−0.390			1.000	

**Table 4:** Results of ANOVAs for CM of specific leaf area (SLA). (**A**) ‘Fixed’ and ‘specific’ averages and intraspecific variability effect were analyzed separately (one-way ANOVA), followed by post hoc (HSD) tests for pairwise comparisons. (**B**) Variability of individual components of trait variation (turnover, intraspecific and their covariation) between belts. (**C**) Proportions of variability of individual components and their parts explained by elevation (belt). For details, see Table 1.

**(A)**															
	**Fixed CM**					**Specific CM**					**Intraspecific CM**				
	**SS**	**DF**	**MS**	**F**	***p***	**SS**	**DF**	**MS**	**F**	***p***	**SS**	**DF**	**MS**	**F**	***p***
Belt	22.120	3	7.373	15.200	<0.0001	41.030	3	13.677	26.890	<0.0001	13.979	3	4.660	170.000	<0.0001
Residuals	19.890	41	0.485			20.850	41	0.509			1.124	41	0.027		
Post hoc (HSD) tests						Post hoc (HSD) tests					Post hoc (HSD) tests				
Belt pairwise comparisons	*p*					Belt pairwise comparisons	*p*				Belt pairwise comparisons	*p*			
1 vs. 2	<0.001					1 vs. 2	<0.001				1 vs. 2	1.000			
1 vs. 3	0.247					1 vs. 3	0.068				1 vs. 3	<0.0001			
1 vs. 4	0.378					1 vs. 4	0.004				1 vs. 4	<0.0001			
2 vs. 3	0.069					2 vs. 3	<0.0001				2 vs. 3	<0.0001			
2 vs. 4	<0.0001					2 vs. 4	<0.0001				2 vs. 4	<0.0001			
3 vs. 4	0.002					3 vs. 4	0.756				3 vs. 4	<0.0001			
**(B)**															
	**Turnover**					**Intraspecific variability**					**Covariation**			**Total**	
Belt	22.120					13.979					4.931			41.030	
Residuals	19.890					1.124					−0.164			20.850	
Total	42.010					15.103					4.767			61.880	
**(C)**															
	**Turnover**					**Intraspecific variability**					**Covariation**			**Total**	
Belt	0.357					0.226					0.080			0.663	
Residuals	0.321					0.018					−0.003			0.337	
Total	0.679					0.244					0.077			1.000	

**Table 5:** Results of ANOVAs for CWM of seed mass (SM). (**A**) ‘Fixed’ and ‘specific’ averages and intraspecific variability effect were analyzed separately (one-way ANOVA), followed by post hoc (HSD) tests for pairwise comparisons. (**B**) Variability of individual components of trait variation (turnover, intraspecific and their covariation) between belts. (**C**) Proportions of variability of individual components and their parts explained by elevation (belt). For details, see Table 1.

**(A)**															
	**Fixed CWM**					**Specific CWM**					**Intraspecific CWM**				
	**SS**	**DF**	**MS**	**F**	***p***	**SS**	**DF**	**MS**	**F**	***p***	**SS**	**DF**	**MS**	**F**	***p***
Belt	7.503	3	2.501	5.278	0.004	7.363	3	2.454	4.974	0.005	2.598	3	0.866	33.410	<0.0001
Residuals	19.429	41	0.474			20.229	41	0.493			1.063	41	0.026		
Post hoc (HSD) tests						Post hoc (HSD) tests					Post hoc (HSD) tests				
Belt pairwise comparisons	*p*					Belt pairwise comparisons	*p*				Belt pairwise comparisons	*p*			
1 vs. 2	1.000					1 vs. 2	0.167				1 vs. 2	<0.0001			
1 vs. 3	0.488					1 vs. 3	0.271				1 vs. 3	0.244			
1 vs. 4	0.158					1 vs. 4	0.825				1 vs. 4	<0.0001			
2 vs. 3	0.467					2 vs. 3	0.988				2 vs. 3	<0.0001			
2 vs. 4	0.136					2 vs. 4	0.012				2 vs. 4	<0.001			
3 vs. 4	0.002					3 vs. 4	0.023				3 vs. 4	0.005			
**(B)**															
	**Turnover**					**Intraspecific variability**					**Covariation**			**Total**	
Belt	7.503					2.598					−2.738			7.363	
Residuals	19.429					1.063					−0.263			20.229	
Total	26.932					3.661					−3.001			27.592	
**(C)**															
	**Turnover**					**Intraspecific variability**					**Covariation**			**Total**	
Belt	0.272					0.094					−0.099			0.267	
Residuals	0.704					0.039					−0.010			0.733	
Total	0.976					0.133					−0.109			1.000	

**Table 6:** Results of ANOVAs for CM of seed mass. (**A**) ‘Fixed’ and ‘specific’ averages and intraspecific variability effect were analyzed separately (one-way ANOVA), followed by post hoc (HSD) tests for pairwise comparisons. (**B**) Variability of individual components of trait variation (turnover, intraspecific and their covariation) between belts. (**C**) Proportions of variability of individual components and their parts explained by elevation (belt). For details, see Table 1.

**(A)**															
	**Fixed CM**					**Specific CM**					**Intraspecific CM**				
	**SS**	**DF**	**MS**	**F**	***p***	**SS**	**DF**	**MS**	**F**	***p***	**SS**	**DF**	**MS**	**F**	***p***
Belt	13.063	3	4.354	23.930	<0.0001	14.557	3	4.852	24.050	<0.0001	3.213	3	1.071	63.060	<0.0001
Residuals	7.459	41	0.182			8.271	41	0.202			0.696	41	0.017		
Post hoc (HSD) tests						Post hoc (HSD) tests					Post hoc (HSD) tests				
Belt pairwise comparisons	*p*					Belt pairwise comparisons	*p*				Belt pairwise comparisons	*p*			
1 vs. 2	0.975					1 vs. 2	<0.001				1 vs. 2	<0.0001			
1 vs. 3	1.000					1 vs. 3	0.430				1 vs. 3	<0.0001			
1 vs. 4	<0.0001					1 vs. 4	0.010				1 vs. 4	<0.0001			
2 vs. 3	0.964					2 vs. 3	0.028				2 vs. 3	<0.0001			
2 vs. 4	<0.0001					2 vs. 4	<0.0001				2 vs. 4	<0.0001			
3 vs. 4	<0.0001					3 vs. 4	<0.0001				3 vs. 4	0.009			
**(B)**															
	**Turnover**					**Intraspecific variability**					**Covariation**			**Total**	
Belt	13.063					3.213					−1.719			14.557	
Residuals	7.459					0.696					0.116			8.271	
Total	20.522					3.909					−1.603			22.828	
**(C)**															
	**Turnover**					**Intraspecific variability**					**Covariation**			**Total**	
Belt	0.572					0.141					−0.075			0.638	
Residuals	0.327					0.030					0.005			0.362	
Total	0.899					0.171					−0.070			1.000	

## Data Availability

All data used in this study are provided as Supplementary Materials (Tables S1–S3).

## Supplementary Materials

The following are available online at https://www.mdpi.com/2223-7747/10/2/359/s1. Supplementary Files: Supplementary Tables S1–S13, Supplementary Figures S1 and S2.
