# Supplementary material for: The Role of Inter- and Intraspecific Variations in Grassland Plant Functional Traits along an Elevational Gradient in a Mediterranean Mountain Area
Source: Plants (Basel). 2021 Feb 13;10(2):359. doi: 10.3390/plants10020359 (PMC7917719; doi:10.3390/plants10020359)
Supplement: Supplementary file 1 [file plants-10-00359-s001.pdf]

**Table S1.** Relative cover of each species in each plot sampled along an elevational gradient in Central Italy (Mount Velino).

| | Belt 1 | | | | | | | | | Belt 2 | | | | | | | | | |
|---|---|---|---|---|---|---|---|---|---|---|---|---|---|---|---|---|---|---|---|
| **Plot number** | **1** | **2** | **3** | **4** | **5** | **6** | **7** | **8** | **9** | **10** | **11** | **12** | **13** | **14** | **15** | **16** | **17** | **18** | **19** |
| Species | | | | | | | | | | | | | | | | | | | |
| *Anthyllis montana* | 0.000 | 0.000 | 0.000 | 0.000 | 0.001 | 0.092 | 0.000 | 0.000 | 0.000 | 0.000 | 0.000 | 0.039 | 0.002 | 0.077 | 0.000 | 0.066 | 0.000 | 0.071 | 0.000 |
| *Brachypodium rupestre* | 0.002 | 0.143 | 0.000 | 0.000 | 0.107 | 0.092 | 0.000 | 0.000 | 0.067 | 0.000 | 0.000 | 0.000 | 0.000 | 0.000 | 0.000 | 0.000 | 0.000 | 0.000 | 0.000 |
| *Brachypodium genuense* | 0.000 | 0.000 | 0.000 | 0.000 | 0.000 | 0.000 | 0.000 | 0.000 | 0.000 | 0.000 | 0.000 | 0.000 | 0.000 | 0.000 | 0.059 | 0.000 | 0.071 | 0.000 | 0.124 |
| *Bromus erectus* | 0.150 | 0.571 | 0.127 | 0.439 | 0.213 | 0.023 | 0.000 | 0.032 | 0.000 | 0.080 | 0.033 | 0.078 | 0.055 | 0.077 | 0.117 | 0.003 | 0.071 | 0.000 | 0.000 |
| *Carex humilis* | 0.150 | 0.000 | 0.254 | 0.281 | 0.027 | 0.092 | 0.235 | 0.129 | 0.067 | 0.080 | 0.267 | 0.494 | 0.055 | 0.153 | 0.059 | 0.133 | 0.000 | 0.285 | 0.000 |
| *Carex macrolepis* | 0.000 | 0.143 | 0.000 | 0.000 | 0.466 | 0.000 | 0.000 | 0.000 | 0.000 | 0.000 | 0.000 | 0.000 | 0.000 | 0.000 | 0.000 | 0.000 | 0.000 | 0.000 | 0.000 |
| *Cytisus spinosus* | 0.038 | 0.143 | 0.032 | 0.035 | 0.027 | 0.023 | 0.029 | 0.032 | 0.067 | 0.040 | 0.033 | 0.039 | 0.111 | 0.153 | 0.059 | 0.066 | 0.000 | 0.000 | 0.124 |
| *Festuca ovina* | 0.038 | 0.000 | 0.032 | 0.035 | 0.000 | 0.000 | 0.235 | 0.258 | 0.000 | 0.000 | 0.000 | 0.000 | 0.000 | 0.000 | 0.000 | 0.000 | 0.000 | 0.000 | 0.000 |
| *Globularia meridionalis* | 0.470 | 0.000 | 0.396 | 0.035 | 0.027 | 0.092 | 0.029 | 0.032 | 0.267 | 0.320 | 0.267 | 0.078 | 0.055 | 0.153 | 0.117 | 0.066 | 0.571 | 0.071 | 0.248 |
| *Heliantemum oelandicum* | 0.150 | 0.000 | 0.127 | 0.140 | 0.107 | 0.184 | 0.235 | 0.129 | 0.267 | 0.080 | 0.067 | 0.078 | 0.111 | 0.077 | 0.059 | 0.133 | 0.286 | 0.285 | 0.005 |
| *Polygala major* | 0.000 | 0.000 | 0.000 | 0.000 | 0.000 | 0.000 | 0.000 | 0.000 | 0.000 | 0.040 | 0.033 | 0.039 | 0.055 | 0.003 | 0.002 | 0.003 | 0.000 | 0.003 | 0.005 |
| *Sesleria juncifolia* | 0.000 | 0.000 | 0.032 | 0.035 | 0.027 | 0.402 | 0.000 | 0.129 | 0.267 | 0.320 | 0.267 | 0.078 | 0.443 | 0.153 | 0.469 | 0.531 | 0.000 | 0.285 | 0.248 |
| *Thymus striatus* | 0.002 | 0.000 | 0.001 | 0.000 | 0.000 | 0.001 | 0.235 | 0.258 | 0.000 | 0.040 | 0.033 | 0.078 | 0.111 | 0.153 | 0.059 | 0.000 | 0.000 | 0.000 | 0.248 |
| Continued | | | | | | | | | | | | | | | | | | | |

**Table S1.** Relative cover of each species in each plot sampled along an elevational gradient in Central Italy (Mount Velino).

| | Belt 3 | | | | | | | | | | | Belt 4 | | | | | | | | | | | | | | |
|---|---|---|---|---|---|---|---|---|---|---|---|---|---|---|---|---|---|---|---|---|---|---|---|---|---|---|
| **Plot number** | **20** | **21** | **22** | **23** | **24** | **25** | **26** | **27** | **28** | **29** | **30** | **31** | **32** | **33** | **34** | **35** | **36** | **37** | **38** | **39** | **40** | **41** | **42** | **43** | **44** | **45** |
| Species | | | | | | | | | | | | | | | | | | | | | | | | | | |
| *Anthyllis montana* | 0.000 | 0.000 | 0.000 | 0.000 | 0.000 | 0.000 | 0.000 | 0.000 | 0.000 | 0.000 | 0.000 | 0.000 | 0.082 | 0.000 | 0.000 | 0.000 | 0.067 | 0.000 | 0.055 | 0.000 | 0.000 | 0.028 | 0.063 | 0.000 | 0.000 | 0.000 |
| *Anthyllis vulneraria* | 0.000 | 0.000 | 0.000 | 0.000 | 0.000 | 0.000 | 0.000 | 0.000 | 0.000 | 0.000 | 0.000 | 0.000 | 0.003 | 0.000 | 0.077 | 0.002 | 0.067 | 0.000 | 0.055 | 0.055 | 0.119 | 0.113 | 0.000 | 0.000 | 0.071 | 0.141 |
| *Avenula praetutiana* | 0.000 | 0.000 | 0.000 | 0.000 | 0.000 | 0.000 | 0.000 | 0.000 | 0.000 | 0.000 | 0.000 | 0.000 | 0.082 | 0.000 | 0.000 | 0.000 | 0.067 | 0.000 | 0.000 | 0.002 | 0.000 | 0.113 | 0.000 | 0.000 | 0.071 | 0.006 |
| *Brachipodium genuense* | 0.200 | 0.000 | 0.143 | 0.071 | 0.091 | 0.000 | 0.038 | 0.200 | 0.119 | 0.148 | 0.000 | 0.000 | 0.000 | 0.000 | 0.000 | 0.000 | 0.000 | 0.000 | 0.000 | 0.000 | 0.000 | 0.000 | 0.000 | 0.000 | 0.000 | 0.000 |
| *Bromus erectus* | 0.050 | 0.000 | 0.036 | 0.285 | 0.091 | 0.066 | 0.002 | 0.200 | 0.030 | 0.037 | 0.495 | 0.000 | 0.082 | 0.164 | 0.308 | 0.000 | 0.267 | 0.000 | 0.055 | 0.055 | 0.030 | 0.000 | 0.000 | 0.000 | 0.000 | 0.000 |
| *Carex humilis* | 0.000 | 0.219 | 0.286 | 0.071 | 0.091 | 0.066 | 0.153 | 0.000 | 0.030 | 0.037 | 0.005 | 0.000 | 0.000 | 0.000 | 0.000 | 0.000 | 0.000 | 0.000 | 0.000 | 0.000 | 0.000 | 0.000 | 0.000 | 0.000 | 0.000 | 0.000 |
| *Carex kitaibeliana* | 0.000 | 0.000 | 0.000 | 0.000 | 0.000 | 0.266 | 0.000 | 0.000 | 0.000 | 0.000 | 0.495 | 0.610 | 0.003 | 0.000 | 0.000 | 0.554 | 0.000 | 0.163 | 0.222 | 0.221 | 0.373 | 0.225 | 0.250 | 0.000 | 0.286 | 0.000 |
| *Carex macrolepis* | 0.050 | 0.000 | 0.036 | 0.071 | 0.000 | 0.000 | 0.002 | 0.000 | 0.030 | 0.148 | 0.000 | 0.000 | 0.000 | 0.000 | 0.000 | 0.000 | 0.000 | 0.000 | 0.000 | 0.000 | 0.000 | 0.000 | 0.000 | 0.000 | 0.000 | 0.000 |
| *Cerastium tomentosum* | 0.000 | 0.000 | 0.000 | 0.000 | 0.000 | 0.000 | 0.000 | 0.000 | 0.000 | 0.000 | 0.000 | 0.000 | 0.000 | 0.007 | 0.077 | 0.002 | 0.067 | 0.007 | 0.002 | 0.055 | 0.030 | 0.001 | 0.000 | 0.019 | 0.071 | 0.000 |
| *Cytisus spinosus* | 0.050 | 0.110 | 0.036 | 0.285 | 0.000 | 0.000 | 0.153 | 0.000 | 0.000 | 0.000 | 0.000 | 0.000 | 0.000 | 0.000 | 0.000 | 0.000 | 0.000 | 0.000 | 0.000 | 0.000 | 0.000 | 0.000 | 0.000 | 0.000 | 0.000 | 0.000 |
| *Festuca striata* | 0.200 | 0.000 | 0.000 | 0.000 | 0.091 | 0.000 | 0.000 | 0.000 | 0.030 | 0.000 | 0.000 | 0.000 | 0.000 | 0.000 | 0.000 | 0.000 | 0.000 | 0.000 | 0.000 | 0.000 | 0.000 | 0.000 | 0.000 | 0.000 | 0.000 | 0.000 |
| *Festuca violacea* | 0.000 | 0.000 | 0.000 | 0.000 | 0.000 | 0.000 | 0.000 | 0.000 | 0.000 | 0.000 | 0.000 | 0.000 | 0.000 | 0.000 | 0.000 | 0.000 | 0.000 | 0.007 | 0.055 | 0.002 | 0.119 | 0.352 | 0.000 | 0.000 | 0.071 | 0.565 |
| *Globularia meridionalis* | 0.000 | 0.342 | 0.036 | 0.003 | 0.091 | 0.066 | 0.153 | 0.000 | 0.521 | 0.001 | 0.000 | 0.195 | 0.003 | 0.000 | 0.000 | 0.177 | 0.000 | 0.000 | 0.222 | 0.055 | 0.030 | 0.113 | 0.063 | 0.000 | 0.000 | 0.000 |
| *Heliantemum nummularium* | 0.200 | 0.000 | 0.143 | 0.000 | 0.000 | 0.000 | 0.038 | 0.200 | 0.119 | 0.296 | 0.000 | 0.000 | 0.003 | 0.000 | 0.077 | 0.000 | 0.000 | 0.000 | 0.000 | 0.221 | 0.000 | 0.000 | 0.000 | 0.000 | 0.000 | 0.000 |
| *Heliantemum oelandicum* | 0.050 | 0.110 | 0.143 | 0.071 | 0.091 | 0.266 | 0.153 | 0.200 | 0.001 | 0.037 | 0.000 | 0.195 | 0.327 | 0.658 | 0.308 | 0.177 | 0.267 | 0.654 | 0.222 | 0.221 | 0.239 | 0.000 | 0.250 | 0.000 | 0.000 | 0.000 |
| *Pedicularis elegans* | 0.000 | 0.000 | 0.000 | 0.000 | 0.000 | 0.000 | 0.000 | 0.000 | 0.000 | 0.000 | 0.000 | 0.000 | 0.003 | 0.007 | 0.000 | 0.044 | 0.000 | 0.000 | 0.000 | 0.055 | 0.030 | 0.000 | 0.063 | 0.000 | 0.000 | 0.000 |
| *Potentilla apennina* | 0.000 | 0.000 | 0.000 | 0.000 | 0.000 | 0.000 | 0.000 | 0.000 | 0.000 | 0.000 | 0.000 | 0.000 | 0.000 | 0.000 | 0.000 | 0.000 | 0.000 | 0.000 | 0.000 | 0.000 | 0.000 | 0.000 | 0.250 | 0.019 | 0.286 | 0.000 |
| *Potentilla crantzii* | 0.000 | 0.000 | 0.000 | 0.000 | 0.000 | 0.000 | 0.000 | 0.000 | 0.000 | 0.000 | 0.000 | 0.000 | 0.000 | 0.000 | 0.000 | 0.000 | 0.000 | 0.000 | 0.000 | 0.000 | 0.000 | 0.028 | 0.000 | 0.000 | 0.071 | 0.000 |
| *Sesleria juncifolia* | 0.000 | 0.219 | 0.143 | 0.071 | 0.091 | 0.266 | 0.307 | 0.002 | 0.001 | 0.148 | 0.005 | 0.000 | 0.327 | 0.164 | 0.000 | 0.044 | 0.067 | 0.163 | 0.055 | 0.000 | 0.030 | 0.000 | 0.063 | 0.481 | 0.071 | 0.141 |
| *Sesleria nitida* | 0.200 | 0.000 | 0.000 | 0.071 | 0.364 | 0.003 | 0.000 | 0.200 | 0.119 | 0.148 | 0.000 | 0.000 | 0.000 | 0.000 | 0.000 | 0.000 | 0.000 | 0.000 | 0.000 | 0.000 | 0.000 | 0.000 | 0.000 | 0.000 | 0.000 | 0.000 |
| *Thymus praecox* | 0.000 | 0.000 | 0.000 | 0.000 | 0.000 | 0.000 | 0.000 | 0.000 | 0.000 | 0.000 | 0.000 | 0.000 | 0.082 | 0.000 | 0.077 | 0.000 | 0.067 | 0.007 | 0.055 | 0.002 | 0.001 | 0.028 | 0.000 | 0.481 | 0.000 | 0.141 |
| *Valeriana montana* | 0.000 | 0.000 | 0.000 | 0.000 | 0.000 | 0.000 | 0.000 | 0.000 | 0.000 | 0.000 | 0.000 | 0.000 | 0.003 | 0.000 | 0.077 | 0.000 | 0.067 | 0.000 | 0.000 | 0.055 | 0.000 | 0.000 | 0.000 | 0.000 | 0.000 | 0.006 |

**Table S2.** Belt-specific values for maximum height (H, cm), specific leaf area (SLA, $mm^2/mg$), and seed mass (SM, mg) for plant species recorded along an elevational gradient in Central Italy (Mount Velino).

| | H | SLA | SM |
|---|---|---|---|
| Belt 1 | | | |
| *Anthyllis montana* | 5.267 | 11.432 | 4.021 |
| *Brachypodium rupestre* | 29.615 | 4.838 | 1.740 |
| *Bromus erectus* | 32.000 | 10.674 | 3.679 |
| *Carex humilis* | 12.933 | 8.974 | 0.758 |
| *Carex macrolepis* | 25.600 | 10.838 | 1.870 |
| *Cytisus spinosus* | 13.460 | 14.290 | 5.659 |
| *Festuca ovina* | 10.833 | 6.048 | 0.812 |
| *Globularia meridionalis* | 3.660 | 8.624 | 0.311 |
| *Heliantemum oelandicum* | 8.730 | 12.009 | 0.745 |
| *Sesleria juncifolia* | 16.400 | 6.940 | 1.638 |
| *Thymus striatus* | 6.567 | 14.043 | 0.077 |
| Belt 2 | | | |
| *Anthyllis montana* | 4.967 | 12.523 | 3.674 |
| *Brachipodium genuense* | 38.533 | 16.581 | 4.353 |
| *Bromus erectus* | 29.250 | 12.464 | 5.455 |
| *Carex humilis* | 14.533 | 9.513 | 0.758 |
| *Cytisus spinosus* | 12.600 | 11.738 | 8.308 |
| *Globularia meridionalis* | 3.430 | 10.519 | 0.210 |
| *Heliantemum oelandicum* | 7.000 | 10.962 | 1.067 |
| *Polygala major* | 9.267 | 13.937 | 3.436 |
| *Sesleria juncifolia* | 24.060 | 5.949 | 3.416 |
| *Thyimus striatus* | 6.000 | 13.834 | 0.119 |
| Belt 3 | | | |
| *Brachypodium genuense* | 34.133 | 16.265 | 3.720 |
| *Bromus erectus* | 18.467 | 8.960 | 2.864 |
| *Carex humilis* | 11.667 | 9.017 | 0.758 |
| *Carex kitabeiliana* | 17.200 | 7.813 | 0.640 |
| *Carex macrolepis* | 29.867 | 9.511 | 2.674 |
| *Cytisus spinosus* | 13.333 | 10.085 | 9.488 |
| *Festuca striata* | 13.000 | 9.863 | 3.683 |
| *Globularia meridionalis* | 3.000 | 9.115 | 0.229 |
| *Heliantemum nummularium* | 23.200 | 12.264 | 1.559 |
| *Heliantemum oelandicum* | 11.260 | 8.720 | 1.015 |
| *Sesleria juncifolia* | 25.933 | 4.283 | 2.040 |
| *Sesleria nitida* | 28.200 | 7.840 | 1.860 |
| Belt 4 | | | |
| *Anthyllis montana* | 5.467 | 8.948 | 2.575 |
| *Anthyllis vulneraria* | 5.000 | 8.861 | 2.610 |
| *Avenula praetutiana* | 4.800 | 5.349 | 1.447 |
| *Bromus erectus* | 25.200 | 13.546 | 4.232 |
| *Carex kitaibeliana* | 13.600 | 8.478 | 1.085 |
| *Cerastium tomentosum* | 5.130 | 12.059 | 0.260 |
| *Festuca violacea* | 8.933 | 10.781 | 0.571 |
| *Globularia meridionalis* | 2.067 | 8.386 | 0.202 |
| *Heliantemum nummularium* | 15.067 | 14.065 | 1.374 |
| *Heliantemum oelandicum* | 7.533 | 10.022 | 0.894 |
| *Pedicularis elegans* | 3.800 | 9.451 | 0.867 |
| *Potentilla apennina* | 4.800 | 7.243 | 0.568 |
| *Potentilla crantzii* | 7.467 | 11.637 | 0.456 |
| *Sesleria juncifolia* | 19.000 | 4.756 | 2.858 |

| | | | |
|---|---|---|---|
| *Sesleria nitida* | 22.533 | 6.873 | 2.693 |
| *Thymus praecox* | 5.600 | 10.043 | 0.131 |
| *Valeriana montana* | 3.727 | 1.879 | 1.350 |

**Table S3.** Fixed values for maximum height (H, cm), specific leaf area (SLA, $mm^2/mg$), and seed mass (SM, mg) for plant species recorded along an elevational gradient in Central Italy (Mount Velino).

| | **H** | **SLA** | **SM** |
|---|---|---|---|
| *Anthyllis montana* | 5.233 | 10.968 | 3.423 |
| *Anthyllis vulneraria* | 5.000 | 8.861 | 2.610 |
| *Avenula praetutiana* | 4.800 | 5.349 | 1.447 |
| *Brachypodium genuense* | 36.333 | 16.423 | 4.036 |
| *Brachypodium rupestre* | 29.615 | 4.838 | 1.740 |
| *Bromus erectus* | 26.229 | 11.411 | 4.057 |
| *Carex humilis* | 13.044 | 9.168 | 0.758 |
| *Carex kitaibeliana* | 15.400 | 8.145 | 0.862 |
| *Carex macrolepis* | 27.733 | 10.175 | 2.272 |
| *Cerastium tomentosum* | 5.130 | 12.059 | 0.260 |
| *Cytisus spinosus* | 13.131 | 12.038 | 7.818 |
| *Festuca ovina* | 10.833 | 6.048 | 0.812 |
| *Festuca striata* | 13.000 | 9.863 | 3.683 |
| *Festuca violacea* | 8.933 | 10.781 | 0.571 |
| *Globularia meridionalis* | 3.039 | 9.161 | 0.238 |
| *Heliantemum nummularium* | 19.133 | 13.165 | 1.466 |
| *Heliantemum oelandicum* | 8.631 | 10.428 | 0.930 |
| *Pedicularis elegans* | 3.800 | 9.451 | 0.867 |
| *Polygala major* | 9.267 | 13.937 | 3.436 |
| *Potentilla apenninica* | 4.800 | 7.243 | 0.568 |
| *Potentilla crantzii* | 7.467 | 11.637 | 0.456 |
| *Sesleria juncifolia* | 21.348 | 5.482 | 2.488 |
| *Sesleria nitida* | 25.367 | 7.356 | 2.277 |
| *Thymus praecox* | 5.600 | 10.043 | 0.131 |
| *Thymus striatus* | 6.283 | 13.939 | 0.098 |
| *Valeriana montana* | 3.727 | 1.879 | 1.350 |

**Table S4.** Specific, fixed and intraspecific CWM values for maximum height (H, cm), specific leaf area (SLA, $mm^2/mg$), and seed mass (SM, mg) in each plot sampled along an elevational gradient in Central Italy (Mount Velino).

| | | H | | | SLA | | | SM | | |
|---|---|---|---|---|---|---|---|---|---|---|
| **Plot number** | **Belt** | **Specific CWM** | **Fixed CWM** | **Intraspecific CWM** | **Specific CWM** | **Fixed CWM** | **Intraspecific CWM** | **Specific CWM** | **Fixed CWM** | **Intraspecific CWM** |
| 1 | 1 | 10.765 | 9.594 | 1.171 | 9.616 | 9.683 | -0.067 | 1.172 | 1.303 | -0.131 |
| 2 | 1 | 28.119 | 25.061 | 3.058 | 10.375 | 10.375 | 0.000 | 3.428 | 4.009 | -0.581 |
| 3 | 1 | 11.194 | 10.375 | 0.819 | 9.462 | 9.484 | -0.022 | 1.132 | 1.271 | -0.139 |
| 4 | 1 | 20.464 | 18.072 | 2.392 | 10.139 | 10.186 | -0.047 | 2.228 | 2.523 | -0.295 |
| 5 | 1 | 24.099 | 23.933 | 0.166 | 10.162 | 9.772 | 0.39 | 2.148 | 2.512 | -0.364 |
| 6 | 1 | 13.964 | 15.740 | -1.776 | 8.690 | 7.798 | 0.892 | 1.637 | 2.009 | -0.372 |
| 7 | 1 | 9.705 | 9.594 | 0.111 | 10.328 | 9.931 | 0.397 | 0.738 | 0.847 | -0.109 |
| 8 | 1 | 10.990 | 11.324 | -0.334 | 9.863 | 9.441 | 0.422 | 0.946 | 1.164 | -0.218 |
| 9 | 1 | 11.402 | 12.531 | -1.129 | 9.226 | 8.414 | 0.812 | 1.262 | 1.663 | -0.401 |
| 10 | 2 | 13.964 | 12.794 | 1.170 | 9.484 | 8.770 | 0.714 | 2.218 | 1.786 | 0.432 |
| 11 | 2 | 13.583 | 12.388 | 1.195 | 9.397 | 8.750 | 0.647 | 1.816 | 1.503 | 0.313 |
| 12 | 2 | 13.646 | 12.618 | 1.028 | 10.375 | 9.886 | 0.489 | 1.774 | 1.552 | 0.222 |
| 13 | 2 | 16.634 | 15.453 | 1.181 | 9.290 | 8.913 | 0.377 | 3.119 | 2.564 | 0.555 |
| 14 | 2 | 12.474 | 11.830 | 0.644 | 10.715 | 10.186 | 0.529 | 2.754 | 2.404 | 0.350 |
| 15 | 2 | 19.770 | 18.030 | 1.704 | 9.204 | 8.650 | 0.554 | 3.133 | 2.483 | 0.650 |
| 16 | 2 | 17.100 | 15.704 | 1.396 | 8.241 | 7.709 | 0.532 | 2.884 | 2.323 | 0.561 |
| 17 | 2 | 8.810 | 8.670 | 0.140 | 11.220 | 10.209 | 1.011 | 1.125 | 0.979 | 0.146 |
| 18 | 2 | 13.614 | 12.882 | 0.732 | 9.204 | 8.610 | 0.594 | 1.778 | 1.459 | 0.319 |
| 19 | 2 | 14.689 | 13.804 | 0.885 | 11.117 | 10.715 | 0.402 | 2.518 | 2.188 | 0.330 |

Continued

**Table S4.** Specific, fixed and intraspecific CWM values for maximum height (H, cm), specific leaf area (SLA, $mm^2/mg$), and seed mass (SM, mg) in each plot sampled along an elevational gradient in Central Italy (Mount Velino).

| | | H | | | SLA | | | SM | | |
|---|---|---|---|---|---|---|---|---|---|---|
| **Plot number** | **Belt** | **Specific CWM** | **Fixed CWM** | **Intraspecific CWM** | **Specific CWM** | **Fixed CWM** | **Intraspecific CWM** | **Specific CWM** | **Fixed CWM** | **Intraspecific CWM** |
| 20 | 3 | 23.335 | 22.542 | 0.793 | 11.117 | 11.561 | -0.444 | 2.965 | 3.048 | -0.083 |
| 21 | 3 | 11.967 | 10.965 | 1.002 | 8.091 | 8.810 | -0.719 | 1.841 | 1.750 | 0.091 |
| 22 | 3 | 19.143 | 18.450 | 0.693 | 9.863 | 10.641 | -0.778 | 1.950 | 2.004 | -0.054 |
| 23 | 3 | 19.099 | 20.654 | -1.555 | 9.419 | 10.914 | -1.495 | 4.375 | 4.295 | 0.080 |
| 24 | 3 | 20.941 | 20.277 | 0.664 | 8.872 | 9.204 | -0.332 | 1.977 | 2.301 | -0.324 |
| 25 | 3 | 16.749 | 14.962 | 1.787 | 7.362 | 8.395 | -1.033 | 1.245 | 1.479 | -0.234 |
| 26 | 3 | 16.255 | 14.555 | 1.700 | 8.110 | 9.099 | -0.989 | 2.600 | 2.477 | 0.123 |
| 27 | 3 | 23.067 | 23.121 | -0.054 | 10.789 | 11.749 | -0.960 | 2.203 | 2.553 | -0.350 |
| 28 | 3 | 13.964 | 13.646 | 0.318 | 10.209 | 10.399 | -0.190 | 1.271 | 1.374 | -0.103 |
| 29 | 3 | 25.882 | 23.823 | 2.059 | 10.233 | 10.889 | -0.656 | 2.158 | 2.286 | -0.128 |
| 30 | 3 | 17.824 | 20.797 | -2.973 | 8.375 | 9.750 | -1.375 | 1.750 | 2.449 | -0.699 |
| 31 | 4 | 10.162 | 11.668 | -1.506 | 8.770 | 8.790 | -0.020 | 0.875 | 0.753 | 0.122 |
| 32 | 4 | 12.190 | 13.428 | -1.238 | 8.091 | 8.453 | -0.362 | 1.936 | 1.884 | 0.052 |
| 33 | 4 | 12.274 | 13.552 | -1.278 | 9.750 | 9.772 | -0.022 | 1.762 | 1.694 | 0.068 |
| 34 | 4 | 16.482 | 18.155 | -1.673 | 9.333 | 9.162 | 0.171 | 2.275 | 2.094 | 0.181 |
| 35 | 4 | 10.789 | 12.303 | -1.514 | 8.551 | 8.630 | -0.079 | 1.038 | 0.899 | 0.139 |
| 36 | 4 | 11.967 | 12.677 | -0.710 | 9.750 | 9.462 | 0.288 | 2.113 | 2.109 | 0.004 |
| 37 | 4 | 10.375 | 11.776 | -1.401 | 8.933 | 9.247 | -0.314 | 1.236 | 1.161 | 0.075 |
| 38 | 4 | 8.995 | 10.023 | -1.028 | 9.141 | 9.376 | -0.235 | 1.202 | 1.186 | 0.016 |
| 39 | 4 | 10.520 | 12.162 | -1.642 | 10.233 | 9.977 | 0.256 | 1.271 | 1.242 | 0.029 |
| 40 | 4 | 10.186 | 11.246 | -1.060 | 9.333 | 9.290 | 0.043 | 1.247 | 1.159 | 0.088 |
| 41 | 4 | 8.072 | 8.570 | -0.498 | 9.120 | 9.183 | -0.063 | 1.014 | 0.991 | 0.023 |
| 42 | 4 | 8.375 | 9.290 | -0.915 | 8.414 | 8.650 | -0.236 | 1.042 | 1.028 | 0.014 |
| 43 | 4 | 12.023 | 13.152 | -1.129 | 7.482 | 7.834 | -0.352 | 1.452 | 1.276 | 0.176 |
| 44 | 4 | 8.851 | 9.528 | -0.677 | 8.318 | 8.260 | 0.058 | 1.057 | 0.968 | 0.089 |
| 45 | 4 | 9.268 | 9.616 | -0.348 | 9.484 | 9.572 | -0.088 | 1.130 | 1.076 | 0.054 |

**Table S5.** Specific, fixed and intraspecific CWM values for maximum height (H, cm), specific leaf area (SLA, $mm^2/mg$), and seed mass (SM, mg) calculated on log-transformed data.

| | | H | | | SLA | | | SM | | |
|---|---|---|---|---|---|---|---|---|---|---|
| Plot number | Belt | Specific CWM | Fixed CWM | Intraspecific CWM | Specific CWM | Fixed CWM | Intraspecific CWM | Specific CWM | Fixed CWM | Intraspecific CWM |
| 1 | 1 | 0.885 | 0.834 | 0.052 | 0.976 | 0.982 | -0.006 | -0.167 | -0.195 | 0.028 |
| 2 | 1 | 1.433 | 1.387 | 0.046 | 0.998 | 1.000 | -0.002 | 0.504 | 0.560 | -0.056 |
| 3 | 1 | 0.924 | 0.884 | 0.039 | 0.969 | 0.972 | -0.003 | -0.150 | -0.168 | 0.018 |
| 4 | 1 | 1.243 | 1.206 | 0.037 | 1.000 | 1.002 | -0.001 | 0.209 | 0.249 | -0.039 |
| 5 | 1 | 1.342 | 1.340 | 0.002 | 0.994 | 0.977 | 0.017 | 0.269 | 0.333 | -0.064 |
| 6 | 1 | 1.078 | 1.113 | -0.036 | 0.921 | 0.869 | 0.052 | 0.112 | 0.190 | -0.078 |
| 7 | 1 | 0.969 | 0.961 | 0.007 | 0.994 | 0.979 | 0.014 | -0.335 | -0.287 | -0.048 |
| 8 | 1 | 1.003 | 1.007 | -0.004 | 0.969 | 0.948 | 0.021 | -0.289 | -0.224 | -0.065 |
| 9 | 1 | 0.973 | 0.980 | -0.007 | 0.948 | 0.907 | 0.041 | -0.054 | -0.002 | -0.053 |
| 10 | 2 | 1.005 | 0.973 | 0.032 | 0.958 | 0.922 | 0.036 | 0.027 | -0.020 | 0.046 |
| 11 | 2 | 1.021 | 0.986 | 0.035 | 0.958 | 0.926 | 0.032 | -0.027 | -0.061 | 0.034 |
| 12 | 2 | 1.071 | 1.047 | 0.025 | 1.007 | 0.985 | 0.022 | -0.005 | -0.035 | 0.030 |
| 13 | 2 | 1.145 | 1.128 | 0.017 | 0.941 | 0.918 | 0.023 | 0.267 | 0.182 | 0.084 |
| 14 | 2 | 0.994 | 0.982 | 0.012 | 1.016 | 0.992 | 0.024 | 0.062 | 0.016 | 0.047 |
| 15 | 2 | 1.207 | 1.174 | 0.033 | 0.935 | 0.905 | 0.030 | 0.290 | 0.205 | 0.085 |
| 16 | 2 | 1.160 | 1.137 | 0.023 | 0.896 | 0.865 | 0.031 | 0.328 | 0.246 | 0.081 |
| 17 | 2 | 0.765 | 0.756 | 0.009 | 1.046 | 1.003 | 0.044 | -0.281 | -0.278 | -0.003 |
| 18 | 2 | 1.056 | 1.052 | 0.004 | 0.950 | 0.921 | 0.029 | 0.119 | 0.065 | 0.054 |
| 19 | 2 | 1.008 | 0.987 | 0.021 | 1.021 | 0.999 | 0.022 | -0.069 | -0.118 | 0.049 |

Continued

**Table S5.** Specific, fixed and intraspecific CWM values for maximum height (H, cm), specific leaf area (SLA, $mm^2/mg$), and seed mass (SM, mg) calculated on log-transformed data.

| | | H | | | SLA | | | SM | | |
|---|---|---|---|---|---|---|---|---|---|---|
| Plot number | Belt | Specific CWM | Fixed CWM | Intraspecific CWM | Specific CWM | Fixed CWM | Intraspecific CWM | Specific CWM | Fixed CWM | Intraspecific CWM |
| 20 | 3 | 1.338 | 1.318 | 0.021 | 1.031 | 1.047 | -0.016 | 0.413 | 0.430 | -0.017 |
| 21 | 3 | 0.946 | 0.926 | 0.019 | 0.890 | 0.932 | -0.043 | -0.070 | -0.059 | -0.011 |
| 22 | 3 | 1.226 | 1.208 | 0.019 | 0.965 | 1.006 | -0.041 | 0.163 | 0.172 | -0.009 |
| 23 | 3 | 1.252 | 1.279 | -0.027 | 0.960 | 1.026 | -0.066 | 0.511 | 0.537 | -0.026 |
| 24 | 3 | 1.248 | 1.234 | 0.014 | 0.929 | 0.947 | -0.018 | 0.202 | 0.257 | -0.055 |
| 25 | 3 | 1.175 | 1.123 | 0.052 | 0.849 | 0.910 | -0.062 | 0.013 | 0.072 | -0.059 |
| 26 | 3 | 1.120 | 1.081 | 0.038 | 0.877 | 0.936 | -0.060 | 0.160 | 0.171 | -0.011 |
| 27 | 3 | 1.334 | 1.320 | 0.013 | 1.017 | 1.055 | -0.038 | 0.299 | 0.341 | -0.042 |
| 28 | 3 | 0.916 | 0.912 | 0.004 | 0.998 | 1.005 | -0.006 | -0.170 | -0.148 | -0.023 |
| 29 | 3 | 1.399 | 1.357 | 0.042 | 0.978 | 1.011 | -0.033 | 0.302 | 0.319 | -0.017 |
| 30 | 3 | 1.251 | 1.302 | -0.051 | 0.921 | 0.983 | -0.062 | 0.131 | 0.271 | -0.139 |
| 31 | 4 | 0.924 | 1.001 | -0.077 | 0.942 | 0.942 | 0.000 | -0.123 | -0.167 | 0.044 |
| 32 | 4 | 1.012 | 1.049 | -0.037 | 0.877 | 0.904 | -0.027 | 0.160 | 0.154 | 0.006 |
| 33 | 4 | 1.026 | 1.076 | -0.050 | 0.970 | 0.979 | -0.009 | 0.142 | 0.140 | 0.002 |
| 34 | 4 | 1.135 | 1.174 | -0.039 | 0.937 | 0.936 | 0.001 | 0.237 | 0.210 | 0.027 |
| 35 | 4 | 0.942 | 1.013 | -0.071 | 0.928 | 0.932 | -0.004 | -0.073 | -0.116 | 0.044 |
| 36 | 4 | 0.969 | 0.992 | -0.022 | 0.946 | 0.941 | 0.005 | 0.161 | 0.165 | -0.004 |
| 37 | 4 | 0.983 | 1.039 | -0.056 | 0.937 | 0.955 | -0.019 | 0.037 | 0.022 | 0.015 |
| 38 | 4 | 0.840 | 0.905 | -0.065 | 0.953 | 0.966 | -0.013 | -0.115 | -0.115 | 0.000 |
| 39 | 4 | 0.945 | 1.003 | -0.058 | 0.980 | 0.971 | 0.008 | 0.016 | 0.007 | 0.009 |
| 40 | 4 | 0.957 | 0.998 | -0.041 | 0.965 | 0.963 | 0.002 | 0.013 | -0.020 | 0.033 |
| 41 | 4 | 0.848 | 0.878 | -0.030 | 0.951 | 0.954 | -0.003 | -0.114 | -0.125 | 0.011 |
| 42 | 4 | 0.855 | 0.896 | -0.041 | 0.918 | 0.930 | -0.012 | -0.058 | -0.070 | 0.012 |
| 43 | 4 | 1.001 | 1.026 | -0.024 | 0.845 | 0.874 | -0.030 | -0.221 | -0.250 | 0.029 |
| 44 | 4 | 0.889 | 0.908 | -0.019 | 0.906 | 0.906 | 0.001 | -0.070 | -0.103 | 0.033 |
| 45 | 4 | 0.929 | 0.937 | -0.007 | 0.960 | 0.969 | -0.009 | -0.137 | -0.146 | 0.008 |

**Table S6.** Specific, fixed and intraspecific CM values for maximum height (H, cm), specific leaf area (SLA, $mm^2/mg$), and seed mass (SM, mg) in each plot sampled along an elevational gradient in Central Italy (Mount Velino).

| | | H | | | SLA | | | SM | | |
|---|---|---|---|---|---|---|---|---|---|---|
| **Plot number** | **Belt** | **Specific CM** | **Fixed CM** | **Intraspecific CM** | **Specific CM** | **Fixed CM** | **Intraspecific CM** | **Specific CM** | **Fixed CM** | **Intraspecific CM** |
| 1 | 1 | 14.725 | 13.851 | 0.874 | 9.938 | 9.629 | 0.309 | 1.722 | 2.056 | -0.334 |
| 2 | 1 | 25.169 | 24.177 | 0.992 | 10.160 | 9.615 | 0.545 | 3.237 | 3.972 | -0.735 |
| 3 | 1 | 13.073 | 12.817 | 0.255 | 10.200 | 9.709 | 0.491 | 1.710 | 2.150 | -0.440 |
| 4 | 1 | 14.002 | 13.751 | 0.251 | 9.651 | 9.105 | 0.546 | 1.943 | 2.443 | -0.500 |
| 5 | 1 | 16.407 | 16.445 | -0.038 | 9.847 | 9.297 | 0.550 | 2.269 | 2.636 | -0.367 |
| 6 | 1 | 14.292 | 14.062 | 0.231 | 10.203 | 9.715 | 0.488 | 2.070 | 2.394 | -0.325 |
| 7 | 1 | 9.364 | 9.160 | 0.204 | 10.665 | 10.130 | 0.534 | 1.393 | 1.776 | -0.382 |
| 8 | 1 | 13.073 | 12.817 | 0.255 | 10.200 | 9.709 | 0.491 | 1.710 | 2.150 | -0.440 |
| 9 | 1 | 14.133 | 14.802 | -0.668 | 9.279 | 8.519 | 0.760 | 1.808 | 2.329 | -0.520 |
| 10 | 2 | 13.268 | 12.622 | 0.646 | 11.114 | 10.695 | 0.419 | 2.846 | 2.478 | 0.368 |
| 11 | 2 | 13.268 | 12.622 | 0.646 | 11.114 | 10.695 | 0.419 | 2.846 | 2.478 | 0.368 |
| 12 | 2 | 12.345 | 11.801 | 0.544 | 11.271 | 10.726 | 0.545 | 2.938 | 2.583 | 0.355 |
| 13 | 2 | 12.345 | 11.801 | 0.544 | 11.271 | 10.726 | 0.545 | 2.938 | 2.583 | 0.355 |
| 14 | 2 | 12.345 | 11.801 | 0.544 | 11.271 | 10.726 | 0.545 | 2.938 | 2.583 | 0.355 |
| 15 | 2 | 16.075 | 15.256 | 0.819 | 11.722 | 11.332 | 0.390 | 3.013 | 2.651 | 0.362 |
| 16 | 2 | 13.138 | 12.490 | 0.648 | 10.951 | 10.324 | 0.627 | 3.290 | 2.894 | 0.397 |
| 17 | 2 | 19.553 | 18.558 | 0.995 | 12.631 | 11.856 | 0.776 | 2.771 | 2.315 | 0.456 |
| 18 | 2 | 10.543 | 10.094 | 0.449 | 10.567 | 9.857 | 0.710 | 2.093 | 1.879 | 0.214 |
| 19 | 2 | 14.413 | 14.005 | 0.408 | 11.931 | 11.630 | 0.302 | 2.987 | 2.721 | 0.266 |

Continued

**Table S6.** Specific, fixed and intraspecific CM values for maximum height (H, cm), specific leaf area (SLA, $mm^2/mg$), and seed mass (SM, mg) in each plot sampled along an elevational gradient in Central Italy (Mount Velino).

| | | H | | | SLA | | | SM | | |
|---|---|---|---|---|---|---|---|---|---|---|
| Plot number | Belt | Specific CM | Fixed CM | Intraspecific CM | Specific CM | Fixed CM | Intraspecific CM | Specific CM | Fixed CM | Intraspecific CM |
| 20 | 3 | 21.433 | 21.195 | 0.238 | 10.439 | 11.357 | -0.919 | 3.358 | 3.317 | 0.040 |
| 21 | 3 | 13.039 | 11.839 | 1.200 | 8.244 | 9.255 | -1.011 | 2.706 | 2.446 | 0.259 |
| 22 | 3 | 18.984 | 18.736 | 0.249 | 9.802 | 10.828 | -1.026 | 2.705 | 2.674 | 0.031 |
| 23 | 3 | 19.540 | 19.428 | 0.112 | 9.311 | 10.182 | -0.872 | 2.739 | 2.764 | -0.025 |
| 24 | 3 | 18.208 | 18.374 | -0.166 | 9.258 | 9.912 | -0.654 | 2.021 | 2.308 | -0.287 |
| 25 | 3 | 16.532 | 16.151 | 0.381 | 7.964 | 8.736 | -0.772 | 1.344 | 1.659 | -0.315 |
| 26 | 3 | 18.984 | 18.736 | 0.249 | 9.802 | 10.828 | -1.026 | 2.705 | 2.674 | 0.031 |
| 27 | 3 | 23.532 | 22.840 | 0.692 | 9.722 | 10.711 | -0.989 | 2.176 | 2.542 | -0.366 |
| 28 | 3 | 19.873 | 19.386 | 0.487 | 9.584 | 10.263 | -0.679 | 2.040 | 2.220 | -0.180 |
| 29 | 3 | 20.636 | 20.095 | 0.541 | 9.553 | 10.308 | -0.755 | 1.858 | 2.058 | -0.200 |
| 30 | 3 | 18.317 | 19.005 | -0.689 | 7.518 | 8.552 | -1.033 | 1.575 | 2.041 | -0.466 |
| 31 | 4 | 7.733 | 9.023 | -1.290 | 8.962 | 9.245 | -0.283 | 0.727 | 0.677 | 0.050 |
| 32 | 4 | 10.261 | 11.331 | -1.070 | 8.512 | 8.592 | -0.080 | 1.717 | 1.704 | 0.013 |
| 33 | 4 | 12.133 | 13.028 | -0.895 | 9.967 | 9.766 | 0.201 | 1.822 | 1.720 | 0.102 |
| 34 | 4 | 11.224 | 12.352 | -1.128 | 9.669 | 9.400 | 0.268 | 1.693 | 1.635 | 0.058 |
| 35 | 4 | 9.833 | 10.964 | -1.131 | 8.611 | 8.868 | -0.257 | 1.434 | 1.316 | 0.117 |
| 36 | 4 | 9.051 | 9.522 | -0.471 | 8.385 | 8.498 | -0.113 | 1.817 | 1.855 | -0.038 |
| 37 | 4 | 9.966 | 10.840 | -0.874 | 9.356 | 9.490 | -0.133 | 0.966 | 0.874 | 0.093 |
| 38 | 4 | 9.753 | 10.454 | -0.701 | 9.588 | 9.734 | -0.146 | 1.542 | 1.557 | -0.015 |
| 39 | 4 | 8.371 | 9.119 | -0.747 | 9.410 | 9.228 | 0.182 | 1.252 | 1.232 | 0.019 |
| 40 | 4 | 9.586 | 10.311 | -0.725 | 9.638 | 9.582 | 0.056 | 1.371 | 1.301 | 0.069 |
| 41 | 4 | 6.451 | 6.734 | -0.282 | 9.394 | 9.667 | -0.274 | 1.037 | 1.111 | -0.074 |
| 42 | 4 | 8.038 | 8.893 | -0.855 | 8.183 | 8.697 | -0.514 | 1.293 | 1.339 | -0.047 |
| 43 | 4 | 8.633 | 9.220 | -0.587 | 8.525 | 8.707 | -0.182 | 0.954 | 0.862 | 0.092 |
| 44 | 4 | 8.591 | 9.110 | -0.519 | 8.645 | 8.695 | -0.049 | 1.232 | 1.158 | 0.074 |
| 45 | 4 | 7.843 | 8.235 | -0.391 | 6.945 | 7.066 | -0.121 | 1.494 | 1.433 | 0.062 |

**Table S7.** Specific, fixed and intraspecific CM values for maximum height (H, cm), specific leaf area (SLA, $mm^2/mg$), and seed mass (SM, mg) calculated on log-transformed data.

| | | H | | | SLA | | | SM | | |
|---|---|---|---|---|---|---|---|---|---|---|
| Plot number | Belt | Specific CM | Fixed CM | Intraspecific CM | Specific CM | Fixed CM | Intraspecific CM | Specific CM | Fixed CM | Intraspecific CM |
| 1 | 1 | 1.072 | 1.047 | 0.025 | 0.971 | 0.961 | 0.009 | -0.050 | -0.017 | -0.034 |
| 2 | 1 | 1.378 | 1.363 | 0.016 | 0.976 | 0.958 | 0.018 | 0.458 | 0.525 | -0.067 |
| 3 | 1 | 1.040 | 1.029 | 0.010 | 0.990 | 0.968 | 0.022 | -0.054 | 0.003 | -0.056 |
| 4 | 1 | 1.071 | 1.062 | 0.009 | 0.968 | 0.943 | 0.025 | 0.098 | 0.147 | -0.049 |
| 5 | 1 | 1.119 | 1.115 | 0.004 | 0.975 | 0.950 | 0.024 | 0.210 | 0.250 | -0.040 |
| 6 | 1 | 1.053 | 1.043 | 0.010 | 0.987 | 0.965 | 0.022 | 0.056 | 0.099 | -0.042 |
| 7 | 1 | 0.933 | 0.914 | 0.019 | 1.009 | 0.991 | 0.017 | -0.202 | -0.164 | -0.038 |
| 8 | 1 | 1.040 | 1.029 | 0.010 | 0.990 | 0.968 | 0.022 | -0.054 | 0.003 | -0.056 |
| 9 | 1 | 1.072 | 1.076 | -0.004 | 0.942 | 0.908 | 0.034 | 0.075 | 0.126 | -0.050 |
| 10 | 2 | 1.029 | 1.021 | 0.009 | 1.033 | 1.013 | 0.020 | 0.129 | 0.081 | 0.048 |
| 11 | 2 | 1.029 | 1.021 | 0.009 | 1.033 | 1.013 | 0.020 | 0.129 | 0.081 | 0.048 |
| 12 | 2 | 0.992 | 0.987 | 0.005 | 1.040 | 1.016 | 0.024 | 0.177 | 0.131 | 0.046 |
| 13 | 2 | 0.992 | 0.987 | 0.005 | 1.040 | 1.016 | 0.024 | 0.177 | 0.131 | 0.046 |
| 14 | 2 | 0.992 | 0.987 | 0.005 | 1.040 | 1.016 | 0.024 | 0.177 | 0.131 | 0.046 |
| 15 | 2 | 1.091 | 1.081 | 0.011 | 1.054 | 1.036 | 0.018 | 0.185 | 0.139 | 0.046 |
| 16 | 2 | 1.019 | 1.011 | 0.008 | 1.028 | 1.000 | 0.027 | 0.315 | 0.274 | 0.041 |
| 17 | 2 | 1.108 | 1.099 | 0.009 | 1.094 | 1.063 | 0.031 | 0.181 | 0.140 | 0.042 |
| 18 | 2 | 0.931 | 0.925 | 0.006 | 1.009 | 0.978 | 0.032 | 0.144 | 0.115 | 0.029 |
| 19 | 2 | 1.028 | 1.027 | 0.000 | 1.059 | 1.043 | 0.015 | 0.150 | 0.109 | 0.041 |

*Continued*

**Table S7.** Specific, fixed and intraspecific CM values for maximum height (H, cm), specific leaf area (SLA, $mm^2/mg$), and seed mass (SM, mg) calculated on log-transformed data.

| | | H | | | SLA | | | SM | | |
|---|---|---|---|---|---|---|---|---|---|---|
| **Plot number** | **Belt** | **Specific CM** | **Fixed CM** | **Intraspecific CM** | **Specific CM** | **Fixed CM** | **Intraspecific CM** | **Specific CM** | **Fixed CM** | **Intraspecific CM** |
| 20 | 3 | 1.298 | 1.285 | 0.013 | 1.008 | 1.045 | -0.037 | 0.433 | 0.440 | -0.007 |
| 21 | 3 | 1.027 | 0.996 | 0.031 | 0.898 | 0.952 | -0.054 | 0.107 | 0.103 | 0.004 |
| 22 | 3 | 1.197 | 1.187 | 0.010 | 0.969 | 1.018 | -0.049 | 0.242 | 0.250 | -0.008 |
| 23 | 3 | 1.207 | 1.201 | 0.006 | 0.947 | 0.990 | -0.042 | 0.251 | 0.271 | -0.021 |
| 24 | 3 | 1.172 | 1.170 | 0.002 | 0.942 | 0.977 | -0.034 | 0.177 | 0.220 | -0.042 |
| 25 | 3 | 1.137 | 1.125 | 0.012 | 0.889 | 0.931 | -0.041 | 0.013 | 0.075 | -0.062 |
| 26 | 3 | 1.197 | 1.187 | 0.010 | 0.969 | 1.018 | -0.049 | 0.242 | 0.250 | -0.008 |
| 27 | 3 | 1.347 | 1.322 | 0.025 | 0.953 | 1.003 | -0.050 | 0.301 | 0.350 | -0.049 |
| 28 | 3 | 1.221 | 1.209 | 0.013 | 0.961 | 0.994 | -0.034 | 0.204 | 0.228 | -0.024 |
| 29 | 3 | 1.233 | 1.219 | 0.014 | 0.957 | 0.994 | -0.037 | 0.164 | 0.191 | -0.027 |
| 30 | 3 | 1.246 | 1.263 | -0.017 | 0.858 | 0.917 | -0.059 | 0.113 | 0.205 | -0.092 |
| 31 | 4 | 0.775 | 0.869 | -0.094 | 0.951 | 0.964 | -0.013 | -0.236 | -0.240 | 0.004 |
| 32 | 4 | 0.889 | 0.926 | -0.037 | 0.887 | 0.895 | -0.009 | 0.086 | 0.085 | 0.001 |
| 33 | 4 | 0.969 | 0.995 | -0.025 | 0.973 | 0.974 | -0.001 | 0.077 | 0.065 | 0.012 |
| 34 | 4 | 0.942 | 0.971 | -0.029 | 0.928 | 0.921 | 0.007 | 0.028 | 0.022 | 0.006 |
| 35 | 4 | 0.868 | 0.916 | -0.048 | 0.921 | 0.938 | -0.016 | -0.006 | -0.025 | 0.018 |
| 36 | 4 | 0.856 | 0.868 | -0.012 | 0.866 | 0.876 | -0.010 | 0.076 | 0.083 | -0.007 |
| 37 | 4 | 0.950 | 0.977 | -0.027 | 0.954 | 0.964 | -0.010 | -0.211 | -0.235 | 0.024 |
| 38 | 4 | 0.885 | 0.918 | -0.033 | 0.968 | 0.979 | -0.011 | -0.051 | -0.047 | -0.003 |
| 39 | 4 | 0.820 | 0.854 | -0.033 | 0.931 | 0.926 | 0.005 | -0.084 | -0.084 | 0.000 |
| 40 | 4 | 0.869 | 0.904 | -0.035 | 0.970 | 0.973 | -0.003 | -0.098 | -0.107 | 0.009 |
| 41 | 4 | 0.761 | 0.784 | -0.023 | 0.962 | 0.974 | -0.012 | -0.191 | -0.181 | -0.011 |
| 42 | 4 | 0.800 | 0.845 | -0.045 | 0.902 | 0.929 | -0.027 | -0.021 | -0.014 | -0.007 |
| 43 | 4 | 0.855 | 0.867 | -0.013 | 0.905 | 0.921 | -0.015 | -0.314 | -0.329 | 0.015 |
| 44 | 4 | 0.876 | 0.889 | -0.013 | 0.915 | 0.921 | -0.006 | -0.043 | -0.063 | 0.020 |
| 45 | 4 | 0.822 | 0.830 | -0.008 | 0.777 | 0.787 | -0.010 | 0.006 | -0.004 | 0.010 |

**Table S8.** Results of ANOVAs for CWM of plant height (H) calculated with log-transformed values. A) 'Fixed' and 'specific' averages and intraspecific variability effect were analyzed separately (one-way ANOVA) followed by post hoc (HSD) tests for pairwise comparisons. B) Variability of individual components of trait variation (turnover, intraspecific and their covariation) between belts. C) Proportions of variability of individual components, and their parts explained by elevation (belt). For details, see Table 1.

**A)**

| | **Fixed CWM** | | | | | **Specific CWM** | | | | | **Intraspecific CWM** | | | | |
|---|---|---|---|---|---|---|---|---|---|---|---|---|---|---|---|
| | **SS** | **DF** | **MS** | **F** | ***p*** | **SS** | **DF** | **MS** | **F** | ***p*** | **SS** | **DF** | **MS** | **F** | ***p*** |
| Belt | 0.262 | 3 | 0.087 | 4.666 | 0.007 | 0.410 | 3 | 0.137 | 7.315 | <0.001 | 0.035 | 3 | 0.012 | 21.150 | <0.0001 |
| Residuals | 0.769 | 41 | 0.019 | | | 0.766 | 41 | 0.019 | | | 0.023 | 41 | 0.001 | | |

| Post hoc (HSD) tests | | Post hoc (HSD) tests | | Post hoc (HSD) tests | |
|---|---|---|---|---|---|
| Belt pairwise comparisons | *p* | Belt pairwise comparisons | *p* | Belt pairwise comparisons | *p* |
| 1 vs 2 | 0.803 | 1 vs 2 | 0.849 | 1 vs 2 | 0.946 |
| 1 vs 3 | 0.308 | 1 vs 3 | 0.323 | 1 vs 3 | 0.997 |
| 1 vs 4 | 0.452 | 1 vs 4 | 0.076 | 1 vs 4 | <0.0001 |
| 2 vs 3 | 0.041 | 2 vs 3 | 0.056 | 2 vs 3 | 0.861 |
| 2 vs 4 | 0.953 | 2 vs 4 | 0.355 | 2 vs 4 | <0.0001 |
| 3 vs 4 | 0.005 | 3 vs 4 | <0.001 | 3 vs 4 | <0.0001 |

**B)**

| | **Turnover** | **Intraspecific variability** | **Covariation** | **Total** |
|---|---|---|---|---|
| Belt | 0.262 | 0.035 | 0.113 | 0.410 |
| Residuals | 0.769 | 0.023 | -0.025 | 0.766 |
| Total | 1.031 | 0.058 | 0.088 | 1.176 |

**C)**

| | **Turnover** | **Intraspecific variability** | **Covariation** | **Total** |
|---|---|---|---|---|
| Belt | 0.223 | 0.030 | 0.096 | 0.349 |
| Residuals | 0.653 | 0.019 | -0.021 | 0.651 |
| Total | 0.876 | 0.049 | 0.075 | 1.000 |

**Table S9.** Results of ANOVAs for CWM of specific leaf area (SLA) calculated with log-transformed values. A) 'Fixed' and 'specific' averages and intraspecific variability effect were analyzed separately (one-way ANOVA) followed by post hoc (HSD) tests for pairwise comparisons. B) Variability of individual components of trait variation (turnover, intraspecific and their covariation) between belts. C) Proportions of variability of individual components, and their parts explained by elevation (belt). For details, see Table 1.

**A)**

| | **Fixed CWM** | | | | | **Specific CWM** | | | | | **Intraspecific CWM** | | | | |
|---|---|---|---|---|---|---|---|---|---|---|---|---|---|---|---|
| | **SS** | **DF** | **MS** | **F** | ***p*** | **SS** | **DF** | **MS** | **F** | ***p*** | **SS** | **DF** | **MS** | **F** | ***p*** |
| Belt | 0.015 | 3 | 0.005 | 2.927 | 0.045 | 0.014 | 3 | 0.005 | 2.387 | 0.083 | 0.029 | 3 | 0.010 | 39.940 | <0.0001 |
| Residuals | 0.072 | 41 | 0.002 | | | 0.079 | 41 | 0.002 | | | 0.010 | 41 | 0.000 | | |

| Post hoc (HSD) tests | | Post hoc (HSD) tests | | Post hoc (HSD) tests | |
|---|---|---|---|---|---|
| Belt pairwise comparisons | *p* | Belt pairwise comparisons | *p* | Belt pairwise comparisons | *p* |
| 1 vs 2 | 0.836 | 1 vs 2 | 1.000 | 1 vs 2 | 0.194 |
| 1 vs 3 | 0.476 | 1 vs 3 | 0.503 | 1 vs 3 | <0.0001 |
| 1 vs 4 | 0.730 | 1 vs 4 | 0.146 | 1 vs 4 | 0.009 |
| 2 vs 3 | 0.099 | 2 vs 3 | 0.532 | 2 vs 3 | <0.0001 |
| 2 vs 4 | 0.999 | 2 vs 4 | 0.153 | 2 vs 4 | <0.0001 |
| 3 vs 4 | 0.043 | 3 vs 4 | 0.887 | 3 vs 4 | <0.0001 |

**B)**

| | **Turnover** | **Intraspecific variability** | **Covariation** | **Total** |
|---|---|---|---|---|
| Belt | 0.015 | 0.029 | -0.031 | 0.014 |
| Residuals | 0.072 | 0.010 | -0.003 | 0.079 |
| Total | 0.088 | 0.039 | -0.034 | 0.093 |

**C)**

| | **Turnover** | **Intraspecific variability** | **Covariation** | **Total** |
|---|---|---|---|---|
| Belt | 0.167 | 0.311 | -0.329 | 0.149 |
| Residuals | 0.780 | 0.106 | -0.035 | 0.851 |
| Total | 0.947 | 0.417 | -0.364 | 1.000 |

**Table S10.** Results of ANOVAs for CWM of seed mass (SM) calculated with log-transformed values. A) 'Fixed' and 'specific' averages and intraspecific variability effect were analyzed separately (one-way ANOVA) followed by post hoc (HSD) tests for pairwise comparisons. B) Variability of individual components of trait variation (turnover, intraspecific and their covariation) between belts. C) Proportions of variability of individual components, and their parts explained by elevation (belt). For details, see Table 1.

**A)**

| | **Fixed CWM** | | | | | **Specific CWM** | | | | | **Intraspecific CWM** | | | | |
|---|---|---|---|---|---|---|---|---|---|---|---|---|---|---|---|
| | **SS** | **DF** | **MS** | **F** | ***p*** | **SS** | **DF** | **MS** | **F** | ***p*** | **SS** | **DF** | **MS** | **F** | ***p*** |
| Belt | 0.395 | 3 | 0.132 | 3.337 | 0.029 | 0.248 | 3 | 0.083 | 2.109 | 0.114 | 0.060 | 3 | 0.020 | 22.780 | <0.0001 |
| Residuals | 1.618 | 41 | 0.039 | | | 1.607 | 41 | 0.039 | | | 0.036 | 41 | 0.001 | | |

| Post hoc (HSD) tests | | Post hoc (HSD) tests | | Post hoc (HSD) tests | |
|---|---|---|---|---|---|
| Belt pairwise comparisons | *p* | Belt pairwise comparisons | *p* | Belt pairwise comparisons | *p* |
| 1 vs 2 | 0.987 | 1 vs 2 | 0.911 | 1 vs 2 | <0.0001 |
| 1 vs 3 | 0.270 | 1 vs 3 | 0.255 | 1 vs 3 | 0.997 |
| 1 vs 4 | 0.787 | 1 vs 4 | 0.994 | 1 vs 4 | <0.001 |
| 2 vs 3 | 0.129 | 2 vs 3 | 0.610 | 2 vs 3 | <0.0001 |
| 2 vs 4 | 0.935 | 2 vs 4 | 0.751 | 2 vs 4 | 0.044 |
| 3 vs 4 | 0.019 | 3 vs 4 | 0.096 | 3 vs 4 | <0.001 |

**B)**

| | **Turnover** | **Intraspecific variability** | **Covariation** | **Total** |
|---|---|---|---|---|
| Belt | 0.395 | 0.060 | -0.207 | 0.248 |
| Residuals | 1.618 | 0.036 | -0.048 | 1.607 |
| Total | 2.014 | 0.096 | -0.255 | 1.855 |

**C)**

| | **Turnover** | **Intraspecific variability** | **Covariation** | **Total** |
|---|---|---|---|---|
| Belt | 0.213 | 0.032 | -0.112 | 0.134 |
| Residuals | 0.873 | 0.019 | -0.026 | 0.866 |
| Total | 1.086 | 0.052 | -0.138 | 1.000 |

**Table S11.** Results of ANOVAs for CM of plant eight (H) calculated using log-transformed values. A) 'Fixed' and 'specific' averages and intraspecific variability effect were analyzed separately (one-way ANOVA) followed by post hoc (HSD) tests for pairwise comparisons. B) Variability of individual components of trait variation (turnover, intraspecific and their covariation) between belts. C) Proportions of variability of individual components, and their parts explained by elevation (belt). For details, see Table 1.

**A)**

| | **Fixed CM** | | | | | **Specific CM** | | | | | **Intraspecific CM** | | | | |
|---|---|---|---|---|---|---|---|---|---|---|---|---|---|---|---|
| | **SS** | **DF** | **MS** | **F** | ***p*** | **SS** | **DF** | **MS** | **F** | ***p*** | **SS** | **DF** | **MS** | **F** | ***p*** |
| Belt | 0.603 | 3 | 0.201 | 31.610 | <0.0001 | 0.796 | 3 | 0.265 | 41.770 | <0.0001 | 0.017 | 3 | 0.006 | 28.330 | <0.0001 |
| Residuals | 0.261 | 41 | 0.006 | | | 0.260 | 41 | 0.006 | | | 0.008 | 41 | 0.000 | | |

| Post hoc (HSD) tests | | Post hoc (HSD) tests | | Post hoc (HSD) tests | |
|---|---|---|---|---|---|
| Belt pairwise comparisons | *p* | Belt pairwise comparisons | *p* | Belt pairwise comparisons | *p* |
| 1 vs 2 | 0.358 | 1 vs 2 | 0.300 | 1 vs 2 | 0.914 |
| 1 vs 3 | 0.008 | 1 vs 3 | 0.008 | 1 vs 3 | 1.000 |
| 1 vs 4 | <0.0001 | 1 vs 4 | <0.0001 | 1 vs 4 | <0.0001 |
| 2 vs 3 | <0.0001 | 2 vs 3 | <0.0001 | 2 vs 3 | 0.916 |
| 2 vs 4 | 0.003 | 2 vs 4 | <0.0001 | 2 vs 4 | <0.0001 |
| 3 vs 4 | <0.0001 | 3 vs 4 | <0.0001 | 3 vs 4 | <0.0001 |

**B)**

| | **Turnover** | **Intraspecific variability** | **Covariation** | **Total** |
|---|---|---|---|---|
| Belt | 0.603 | 0.017 | 0.176 | 0.796 |
| Residuals | 0.261 | 0.008 | -0.009 | 0.260 |
| Total | 0.864 | 0.025 | 0.167 | 1.056 |

**C)**

| | **Turnover** | **Intraspecific variability** | **Covariation** | **Total** |
|---|---|---|---|---|
| Belt | 0.571 | 0.016 | 0.166 | 0.754 |
| Residuals | 0.247 | 0.008 | -0.008 | 0.246 |
| Total | 0.818 | 0.024 | 0.158 | 1.000 |

**Table S12.** Results of ANOVAs for CM of specific leaf area (SLA) calculated using log-transformed values. A) 'Fixed' and 'specific' averages and intraspecific variability effect were analyzed separately (one-way ANOVA) followed by post hoc (HSD) tests for pairwise comparisons. B) Variability of individual components of trait variation (turnover, intraspecific and their covariation) between belts. C) Proportions of variability of individual components, and their parts explained by elevation (belt). For details, see Table 1.

**A)**

| | **Fixed CM** | | | | | **Specific CM** | | | | | **Intraspecific CM** | | | | |
|---|---|---|---|---|---|---|---|---|---|---|---|---|---|---|---|
| | **SS** | **DF** | **MS** | **F** | ***p*** | **SS** | **DF** | **MS** | **F** | ***p*** | **SS** | **DF** | **MS** | **F** | ***p*** |
| Belt | 0.053 | 3 | 0.018 | 12.300 | <0.0001 | 0.098 | 3 | 0.033 | 21.540 | <0.0001 | 0.032 | 3 | 0.011 | 182.200 | <0.0001 |
| Residuals | 0.059 | 41 | 0.001 | | | 0.062 | 41 | 0.002 | | | 0.002 | 41 | 0.000 | | |

| Post hoc (HSD) tests | | Post hoc (HSD) tests | | Post hoc (HSD) tests | |
|---|---|---|---|---|---|
| Belt pairwise comparisons | *p* | Belt pairwise comparisons | *p* | Belt pairwise comparisons | *p* |
| 1 vs 2 | 0.005 | 1 vs 2 | 0.004 | 1 vs 2 | 0.946 |
| 1 vs 3 | 0.357 | 1 vs 3 | 0.159 | 1 vs 3 | <0.0001 |
| 1 vs 4 | 0.328 | 1 vs 4 | 0.006 | 1 vs 4 | <0.0001 |
| 2 vs 3 | 0.182 | 2 vs 3 | <0.0001 | 2 vs 3 | <0.0001 |
| 2 vs 4 | <0.0001 | 2 vs 4 | <0.0001 | 2 vs 4 | <0.0001 |
| 3 vs 4 | 0.003 | 3 vs 4 | 0.559 | 3 vs 4 | <0.0001 |

**B)**

| | **Turnover** | **Intraspecific variability** | **Covariation** | **Total** |
|---|---|---|---|---|
| Belt | 0.053 | 0.032 | 0.013 | 0.098 |
| Residuals | 0.059 | 0.002 | 0.001 | 0.062 |
| Total | 0.113 | 0.034 | 0.014 | 0.161 |

**C)**

| | **Turnover** | **Intraspecific variability** | **Covariation** | **Total** |
|---|---|---|---|---|
| Belt | 0.332 | 0.197 | 0.083 | 0.612 |
| Residuals | 0.369 | 0.015 | 0.004 | 0.388 |
| Total | 0.701 | 0.211 | 0.088 | 1.000 |

**Table S13.** Results of ANOVAs for CM of seed mass (SLA) calculated using log-transformed values. A) ‘Fixed’ and ‘specific’ averages and intraspecific variability effect were analyzed separately (one-way ANOVA) followed by post hoc (HSD) tests for pairwise comparisons. B) Variability of individual components of trait variation (turnover, intraspecific and their covariation) between belts. C) Proportions of variability of individual components, and their parts explained by elevation (belt). For details, see Table 1.

**A)**

| | **Fixed CM** | | | | | **Specific CM** | | | | | **Intraspecific CM** | | | | |
|---|---|---|---|---|---|---|---|---|---|---|---|---|---|---|---|
| | **SS** | **DF** | **MS** | **F** | ***p*** | **SS** | **DF** | **MS** | **F** | ***p*** | **SS** | **DF** | **MS** | **F** | ***p*** |
| Belt | 0.643 | 3 | 0.214 | 13.330 | <0.0001 | 0.584 | 3 | 0.195 | 12.260 | <0.0001 | 0.049 | 3 | 0.016 | 61.100 | <0.0001 |
| Residuals | 0.659 | 41 | 0.016 | | | 0.651 | 41 | 0.016 | | | 0.011 | 41 | 0.000 | | |

| Post hoc (HSD) tests | | Post hoc (HSD) tests | | Post hoc (HSD) tests | |
|---|---|---|---|---|---|
| Belt pairwise comparisons | *p* | Belt pairwise comparisons | *p* | Belt pairwise comparisons | *p* |
| 1 vs 2 | 0.972 | 1 vs 2 | 0.199 | 1 vs 2 | <0.0001 |
| 1 vs 3 | 0.133 | 1 vs 3 | 0.067 | 1 vs 3 | 0.096 |
| 1 vs 4 | 0.009 | 1 vs 4 | 0.101 | 1 vs 4 | <0.0001 |
| 2 vs 3 | 0.274 | 2 vs 3 | 0.958 | 2 vs 3 | <0.0001 |
| 2 vs 4 | 0.002 | 2 vs 4 | <0.001 | 2 vs 4 | <0.0001 |
| 3 vs 4 | <0.0001 | 3 vs 4 | <0.0001 | 3 vs 4 | <0.0001 |

**B)**

| | **Turnover** | **Intraspecific variability** | **Covariation** | **Total** |
|---|---|---|---|---|
| Belt | 0.643 | 0.049 | -0.107 | 0.584 |
| Residuals | 0.659 | 0.011 | -0.019 | 0.651 |
| Total | 1.302 | 0.060 | -0.126 | 1.236 |

**C)**

| | **Turnover** | **Intraspecific variability** | **Covariation** | **Total** |
|---|---|---|---|---|
| Belt | 0.520 | 0.040 | -0.087 | 0.473 |
| Residuals | 0.533 | 0.009 | -0.015 | 0.527 |
| Total | 1.053 | 0.048 | -0.102 | 1.000 |

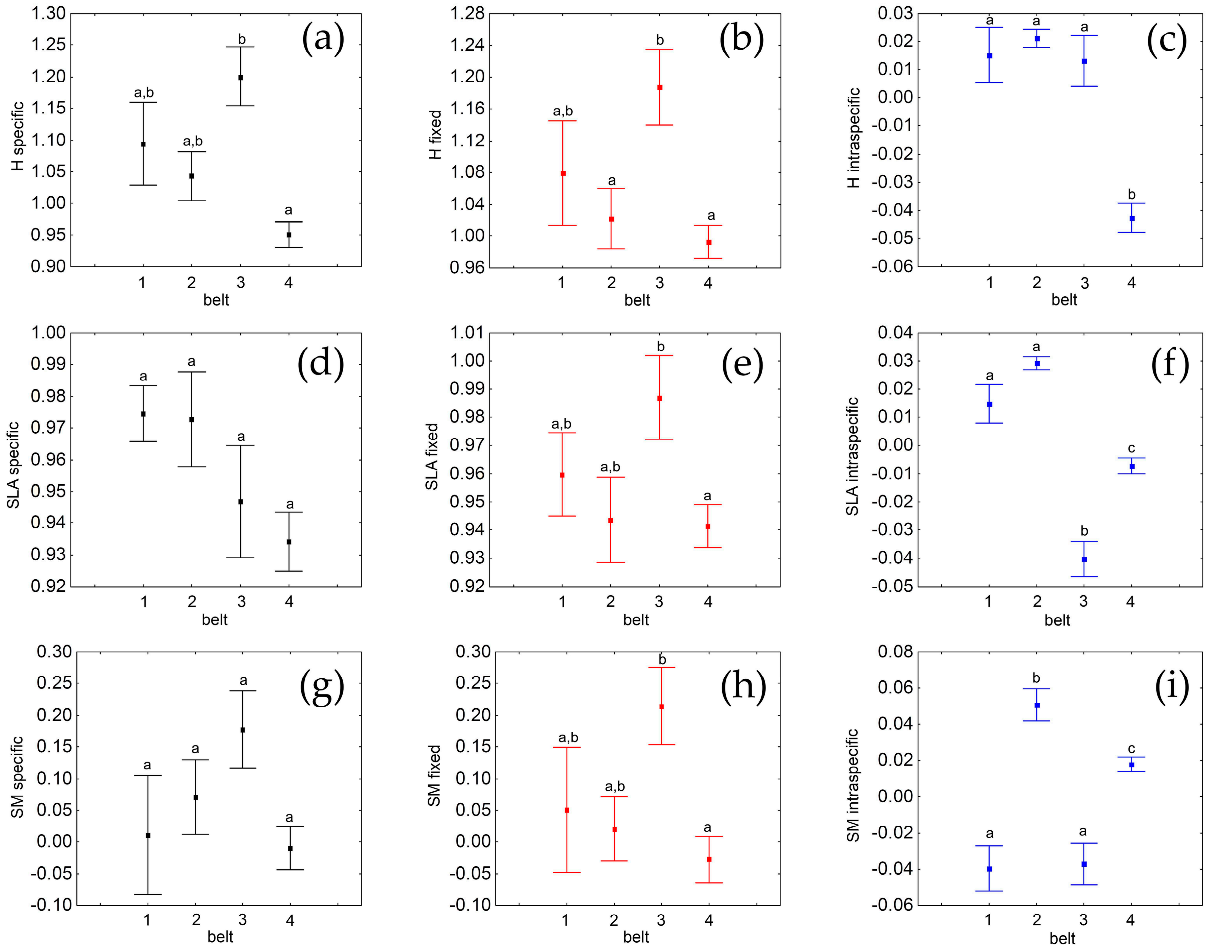


**Figure 1S.** Averages and standard errors of CWM values (after log-transformation) in the four elevational belts (Elevation: 1, 2, 3, 4) calculated for plant eight (H: a, b, c), specific leaf area (SLA: d, e, f) and seed mass (SM, g, h, i), along an elevational gradient in Central Italy. CWMs were calculated for the specific variability (a, d, g), the fixed component (b, e, h) and the intraspecific variability (c, f, i). Letters indicate Tukey honest significant difference (HSD) post-hoc tests at the $p < 0.05$ level, following ANOVA.

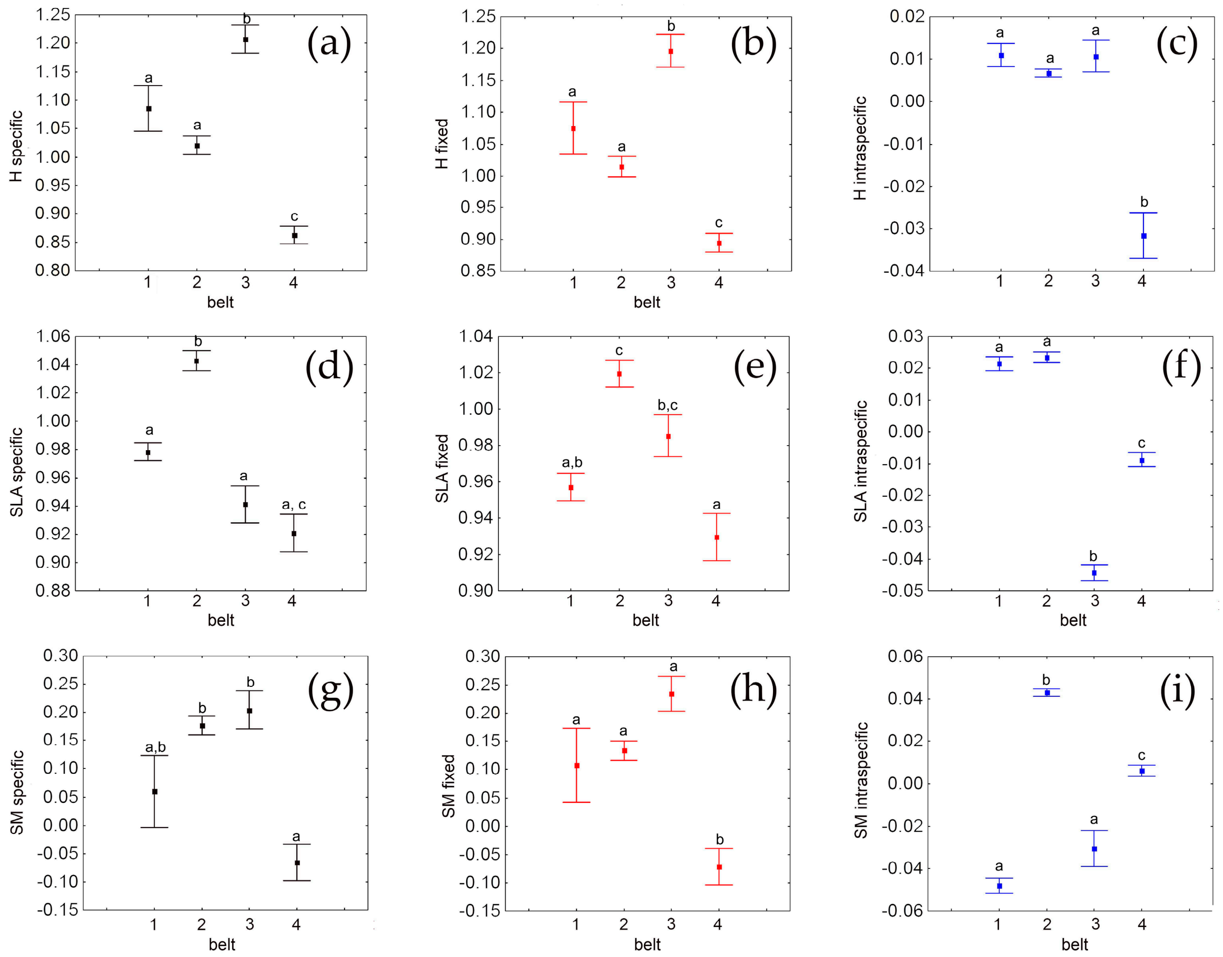


**Figure 2S.** Averages and standard errors of CM values (after log-transformation) in the four elevational belts (Elevation: 1, 2, 3, 4) calculated for plant eight (H: a, b, c), specific leaf area (SLA: d, e, f) and seed mass (SM, g, h, i), along an elevational gradient in Central Italy. CWMs were calculated for the specific variability (a, d, g), the fixed component (b, e, h) and the intraspecific variability (c, f, i). Letters indicate Tukey honest significant difference (HSD) post-hoc tests at the $p < 0.05$ level, following ANOVA.
